# Ancient Dispersal of the Human Fungal Pathogen *Cryptococcus gattii* from the Amazon Rainforest

**DOI:** 10.1371/journal.pone.0071148

**Published:** 2013-08-07

**Authors:** Ferry Hagen, Paulo C. Ceresini, Itzhack Polacheck, Hansong Ma, Filip van Nieuwerburgh, Toni Gabaldón, Sarah Kagan, E. Rhiannon Pursall, Hans L. Hoogveld, Leo J. J. van Iersel, Gunnar W. Klau, Steven M. Kelk, Leen Stougie, Karen H. Bartlett, Kerstin Voelz, Leszek P. Pryszcz, Elizabeth Castañeda, Marcia Lazera, Wieland Meyer, Dieter Deforce, Jacques F. Meis, Robin C. May, Corné H. W. Klaassen, Teun Boekhout

**Affiliations:** 1 Department of Yeast and Basidiomycete Research, CBS-KNAW Fungal Biodiversity Centre, Utrecht, The Netherlands; 2 Department of Medical Microbiology and Infectious Diseases, Canisius-Wilhelmina Hospital, Nijmegen, The Netherlands; 3 Department of Phytopathology, UNESP - University of São Paulo State, Ilha Solteira Campus, Ilha Solteira, Brazil; 4 Department of Clinical Microbiology and Infectious Diseases, Hadassah-Hebrew University Medical Center, Jerusalem, Israel; 5 School of Biosciences, University of Birmingham, Edgbaston, Birmingham, United Kingdom; 6 Laboratory of Pharmaceutical Biotechnology, Faculty of Pharmaceutical Sciences, Ghent University, Ghent, Belgium; 7 Centre for Genomic Regulation (CRG) and UPF Doctor Aiguader, Barcelona, Spain; 8 Netherlands Institute of Ecology (NIOO-KNAW), Centre for Limnology, Wageningen, The Netherlands; 9 Centrum Wiskunde & Informatica (CWI), Amsterdam, The Netherlands; 10 Department of Operations Research, VU University, Amsterdam, The Netherlands; 11 School of Environmental Health, University of British Columbia, Vancouver, British Columbia, Canada; 12 Instituto Nacional de Salud, Grupo de Microbiología, Zona 6 CAN, Bogotá, Colombia; 13 Laboratorio de Micologia, Instituto de Pesquisa Clinica Evandro Chagas, Fundaçao Oswaldo Cruz – FIOCRUZ, Rio de Janeiro, Brazil; 14 Molecular Mycology Research Laboratory, Center for Infectious Diseases and Microbiology, Westmead Millennium Institute, Sydney Emerging Disease and Biosecurity Institute, Sydney Medical School – Westmead Hospital, University of Sydney, Westmead, NSW, Australia; 15 Department of Medical Microbiology, Radboud University Nijmegen Medical Centre, Nijmegen, The Netherlands; 16 Shanghai Key Laboratory of Molecular Medical Mycology, Changzheng Hospital, Second Military Medical University, Shanghai, China; 17 Department of Internal Medicine and Infectious Diseases, University Medical Center, Utrecht, The Netherlands; University of Strasbourg, France

## Abstract

Over the past two decades, several fungal outbreaks have occurred, including the high-profile ‘Vancouver Island’ and ‘Pacific Northwest’ outbreaks, caused by *Cryptococcus gattii*, which has affected hundreds of otherwise healthy humans and animals. Over the same time period, *C. gattii* was the cause of several additional case clusters at localities outside of the tropical and subtropical climate zones where the species normally occurs. In every case, the causative agent belongs to a previously rare genotype of *C. gattii* called AFLP6/VGII, but the origin of the outbreak clades remains enigmatic. Here we used phylogenetic and recombination analyses, based on AFLP and multiple MLST datasets, and coalescence gene genealogy to demonstrate that these outbreaks have arisen from a highly-recombining *C. gattii* population in the native rainforest of Northern Brazil. Thus the modern virulent *C. gattii* AFLP6/VGII outbreak lineages derived from mating events in South America and then dispersed to temperate regions where they cause serious infections in humans and animals.

## Introduction

During the past two decades, fungal outbreaks caused by previously rare genotypes or even novel species have emerged that affect humans, mammals and amphibians. Among them are the white-nose-syndrome in bats due to *Geomyces destructans* [1], the killing of frogs by *Batrachochytrium dendrobatidis* [2], and the ongoing *Cryptococcus gattii* outbreaks in Canada (the so-called ‘Vancouver Island’ outbreak) and the Pacific Northwest of the USA, as well as several case clusters of *C. gattii* infections in Western Australia, and Brazil [3]–[8].

Among the most prominent of these outbreaks is the Vancouver Island outbreak (British Columbia, Canada), caused by *C. gattii*, which expanded from Vancouver Island (Canada) to the mainland of British Columbia and the Pacific Northwest [3], [6], [7]. Until the Vancouver Island outbreak, *C. gattii* was almost exclusively known as the cause of rare cryptococcal infections in otherwise healthy humans and animals living in tropical and subtropical regions, mainly in Australasia and South America [5], [7], [9], [10]. However, over the past decade hundreds of apparently healthy people and animals in the affected Vancouver Island outbreak area have been infected by *C. gattii* strains belonging to the AFLP6/VGII genotypic lineage [4]. Since the onset of this outbreak, the incidence of *C. gattii* infections on Vancouver Island was estimated to be approximately 27 times higher than in Northern Australia where *C. gattii* is endemic [5], [7]. Over this period, 19 patients died due to a *C. gattii* infection, which is a case-fatality rate of 8.7% [5]. Concurrently with the Vancouver Island outbreak, there have been clusters of AFLP6/VGII *C. gattii*-related disease reported from captive psittacine birds in São Paulo, Brazil (this study; [8]), a higher incidence of *C. gattii* AFLP6/VGII infections among immunocompetent children in Northern Brazil [11]–[13] and a case cluster among sheep in Western Australia [4], [14].

All these outbreaks and case clusters are caused by genotype AFLP6/VGII strains, one of the five known genotypes within the *C. gattii* complex [6], [7], [15]–[17]. This genotype AFLP6/VGII has been further differentiated into three major subgenotypes, the so-called ‘major’ genotype AFLP6A/VGIIa, the ‘minor’ genotype AFLP6B/VGIIb, and a novel genotype AFLP6C/VGIIc that emerged in the Pacific Northwest [3], [6], [7], [17]. Comparative virulence studies suggested that *C. gattii* strains of the major genotype AFLP6A/VGIIa have a higher virulence potential than strains that belong to AFLP6B/VGIIb [6], [18], [19], whilst the recently discovered AFLP6C/VGIIc strains from the Pacific Northwest outbreak have a similar virulence as those of genotype AFLP6A/VGIIa strains from the Vancouver Island outbreak [3].

Trees are the natural habitat of *C. gattii* [3], [7], [9], [20]–[25], and soil, trees, water and air in the Vancouver Island outbreak region contain high quantities of *C. gattii* propagules [3], [25]. The recent range expansion of this pathogen to the Canadian mainland is likely caused by traffic-based transport of soil and dust-contaminated individuals and motor vehicles [25].

The molecular epidemiology of the *C. gattii* complex has been extensively studied and led to several hypotheses to explain the origin of the Vancouver Island outbreak [3], [6], [7], [15], [17]. Two hypotheses emerged from these studies, firstly it has been proposed that same-sex mating between low virulent mating-type α strains within the Australasian population or while in transit to North America gave rise to the highly virulent genotype that subsequently caused the Canadian outbreak [6]. The same authors suggested also that the Vancouver Island outbreak may have been originated from other geographic localities. Ngamskulrungroj et al. [17] observed that the South American *C. gattii* population demonstrated the highest genetic diversity and these authors suggested that the origin of this species may be in South America. Thus, the origin of the highly virulent lineages within *C. gattii* genotype AFLP6/VGII remains unclear.

Understanding the genetic and geographic origin of these outbreaks is critical in order to control the spread of *C. gattii*. Here we provide an evolutionary model for the origin of the *C. gattii* epidemics using extensive molecular population analyses that includes amplified fragment length polymorphisms (AFLP) analysis, a novel multi-locus microsatellite panel, and coalescence gene genealogy analysis based on data obtained from a novel multi-locus sequence typing scheme based on sequence characterized AFLP regions (SCAR-MLST). In addition, we performed additional sequencing and re-analyzed recently published MLST data [3], [8] by expanding the geographic range through the inclusion of more strains from South America. These analyses revealed that South American *C. gattii* AFLP6/VGII strains represent the ancestral lineages within the global population, which is in agreement with the observed high genetic diversity at that locality. Coalescence gene genealogy analysis showed that the basal lineages within genotype AFLP6/VGII have a South American origin. Thus we provide strong evidence that South America is the cradle for the global population, including the expanding *C. gattii* AFLP6/VGII outbreaks in British Columbia (Canada) and in the Pacific Northwest of the USA [3], [6], [7].

## Results

### South America is the Origin of the Global *C. gattii* AFLP6/VGII Population

**Figure 1:**
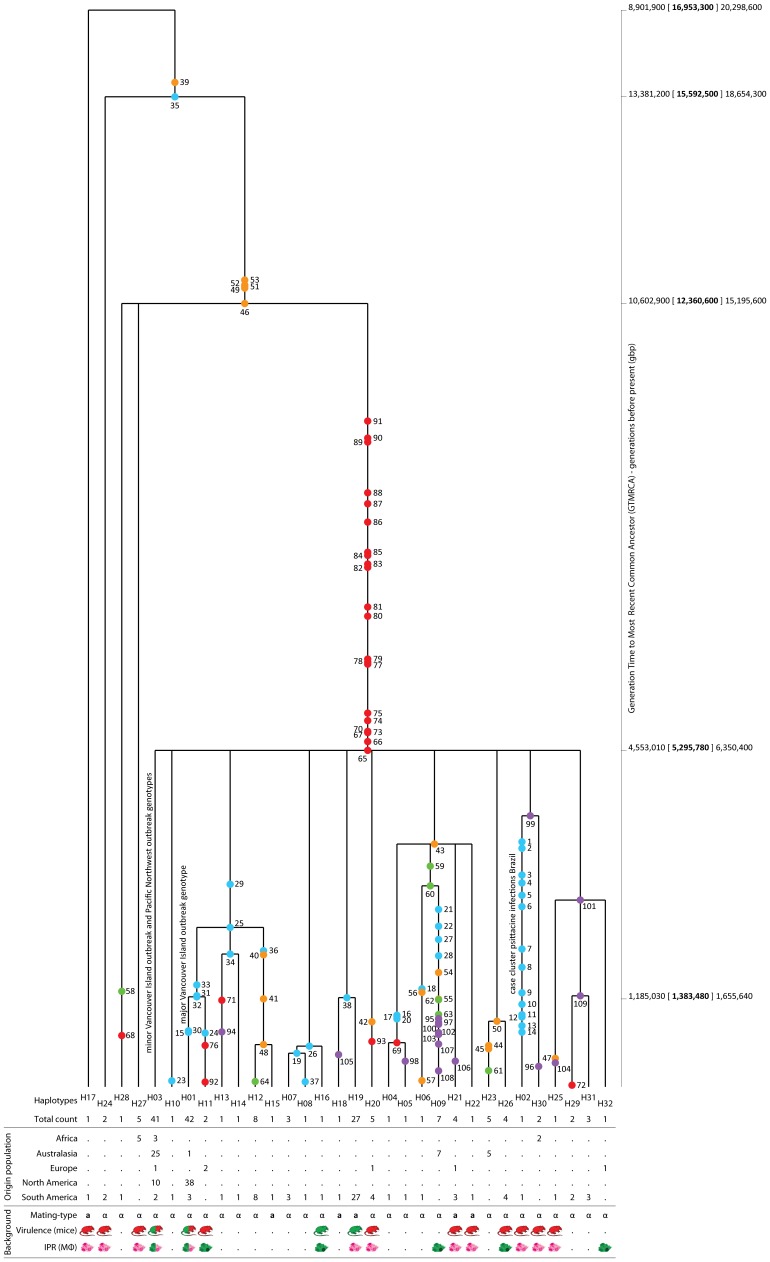
Coalescence gene genealogy based on SCAR-MLST data. The historical coalescence gene genealogy of the global *Cryptococcus gattii* AFLP6/VGII population structure was reconstructed by using five nuclear SCAR-MLST loci from which homoplasious sites were removed resulting in a total number of 109 informative sites indicated on the branches as blue (‘Fragment 15′ locus), orange (‘Fragment 32′ locus), green (‘Fragment 34′ locus), red (‘Fragment 54′ locus) and purple (IGS1 locus) dots. Numbers next to these dots represents the position of the site within the sequence of informative sites (Table S1). Haplotypes that represent lineages currently involved in outbreaks or case cluster are indicated along the lines of these haplotypes. Numbers behind the populations indicate the number of strains within the given population/haplotypes. Mouse virulence and IPR results are shown as red or green mice and macrophages, respectively, to represent virulent and non-virulent strains as indicated in Fig. 4 and 5, and Table 1 and S3. The ‘generation time to the most common recent ancestor’ (GTMRCA) in ‘generations before present’ is provided (bold), including the lower and upper boundaries.

The historical population structure of *C. gattii* AFLP6/VGII was assessed by application of coalescence gene genealogy analysis using the SCAR-MLST dataset which was analyzed after removing 59 homoplasious sites in the 3,357 nucleotides alignment which resulted in 32 haplotypes (indicated as ‘H’ in Fig. 1; Table 1, 2 and S1) compared to 49 sequence types (STs) when all recombination breakpoints remained intact. The most basal lineage within the coalescence gene genealogy was found to be of South American descent and is represented by an environmental strain isolated from a tree in the pristine Brazilian Amazon rainforest (LMM645; haplotype H17, mating-type a). The next diverging lineage is the South American lineage H24 (LMM265 and LMM266; Brazil, mating-type α) (Fig. 1; Table 1). Mutations deep in the coalescence gene genealogy divided the two basal lineages from three younger branches, which were differentiated from each other by more recent mutations. Two of these ancient lineages had an African and South American origin (H27 and H28). The four basal lineages diverged subsequently over time into 28 haplotypes associated with the occurrence of 22 mutations in the ‘Fragment 54′ locus (Fig. 1; Table 1). Compared to the other case cluster and North American outbreak lineages, the psittacine bird *C. gattii* case cluster lineage H02 contained substantially more mutations due to a 20bp region in the non-coding locus ‘Fragment 15′, which is highly polymorphic compared to the other four nuclear SCAR-MLST loci studied (Fig. 1; Table S1).

**Table 1:** Summarized overview of coalescence gene genealogy haplotypes, SCAR-MLST sequence types, mating-types and virulence related data.

Haplotype number^A^	Number ofstrains	Sequence Type (ST) per haplotype (# strains)^B^	Mating-type		Population^C^					MΦ J774 IPR and BALB/c mice data^D^		
			MATa	MATα	AFR	AUS	EUR	NA	SA	Strain (ST-number)^B^	MΦ J774 IPR	ST_50_ survival (# mice)
H01	42	ST01 (19/14/5), ST02 (1/−/−), ST03 (−/1/−), ST12 (−/1/−), ST28 (−/1/−)	–	42	–	1	–	38	3	CBS6956B (ST01)	1.35	24.0 (10)
										CBS10485 (ST01)	1.78	3.9 (10)
										RB59FH (ST01)	1.81	4.0 (10)
										A1M-R265 (ST01)	1.74	4.3 (10)
										RB50 (ST01)	1.87	6.0 (10)
										A1M-F3016 (ST01)	1.47	6.8 (10)
										CBS7750 (ST01)	0.93	avirulent (10)
H02	1	ST31 (−/−/1)	–	1	–	–	–	–	1	LA362 (ST31)	1.39	9.2 (10)
H03	41	ST04 (13/8/6), ST05 (−/1/−), ST06 (1/−/−), ST11 (1/−/−), ST16 (1/−/−), ST39 (3/−/2), ST40 (3/−/−), ST45 (−/−/1), ST46 (1/−/−)	–	41	–	–	–	–	2	CCA242 (ST11)	1.12	10.0 (9)
										A1M-R272 (ST04)	0.88	avirulent (10)
H04	1	ST21 (−/1/−)	–	1	–	–	–	–	1	–	–	–
H05	1	ST22 (−/1/−)	–	1	–	–	–	–	1	–	–	–
H06	1	ST17 (1/−/−)	–	1	–	–	–	–	1	–	–	–
H07	3	ST23 (1/1/−), ST24 (−/1/−)	–	3	–	–	–	–	3	–	–	–
H08	1	ST27 (1/−/−)	–	1	–	–	–	–	1	–	–	–
H09	7	ST44 (7/−/−)	–	7	–	7	–	–	–	–	–	–
H10	1	ST20 (1/−/−)	–	1	–	–	–	–	1	–	–	–
H11	2	ST07 (1/−/−), ST08 (1/−/−)	–	2	–	–	2	–	–	CBS10089 (ST08)	0.44	13.9 (8)
H12	8	ST14 (8/−/−)	–	8	–	–	–	–	8	–	–	–
H13	1	ST29 (1/−/−)	–	1	–	–	–	–	1	–	–	–
H14	1	ST30 (1/−/−)	–	1	–	–	–	–	1	–	–	–
H15	1	ST13 (1/−/−)	1	–	–	–	–	–	1	–	–	–
H16	1	ST10 (−/1−/)	–	1	–	–	–	–	1	CBS8684 (ST10)	0.90	avirulent (8)
H17	1	ST26 (−/1/−)	1	–	–	–	–	–	1	LMM645 (ST26)	2.37	10.0 (9)
H18	1	ST19 (1/−/−)	1	–	–	–	–	–	1	–	–	–
H19	27	ST09 (11/14/1), ST18 (1/−/−)	27	–	–	–	–	–	27	CBS1930 (ST09)	1.14	avirulent (10)
H20	5	ST33 (4/−/−), ST34 (1/−/−)	–	5	–	–	1	–	4	IP04/335 (ST34)	2.75	6.4 (8)
H21	4	ST41 (4/−/−)	4	–	–	–	1	–	3	CBS10090 (ST41)	1.71	6.0 (9)
H22	1	ST42 (1/−/−)	1	–	–	–	–	–	1	LMM261 (ST42)	1.42	6.0 (9)
H23	5	ST15 (5/−/−)	–	5	–	5	–	–	–	–	–	–
H24	2	ST48 (2/−/−)	–	2	–	–	–	–	2	LMM265 (ST48)	2.54	10.0 (9)
H25	1	ST43 (1/−/−)	–	1	–	–	–	–	1	IP97/170 (ST43)	2.82	6.9 (8)
H26	4	ST49 (2/2/−)	–	4	–	–	–	–	4	ICB180 (ST49)	0.42	14.9 (9)
H27	5	ST38 (5/−/−)	–	5	5	–	–	–	–	IP01/935–1 (ST38)	2.92	14.3 (9)
H28	1	ST47 (1/−/−)	–	1	–	–	–	–	1	–	–	–
H29	2	ST35 (−/1/−), ST36 (−/1/−)	–	2	–	–	–	–	2	–	–	–
H30	2	ST37 (2/−/−)	–	2	2	–	–	–	–	IHEM11489S (ST37)	1.77	avirulent (9)
H31	3	ST25 (3/−/−)	–	3	–	–	–	–	3	–	–	–
H32	1	ST32 (1/−/−)	–	1	–	–	1	–	–	–	–	–

**Figure 2:**
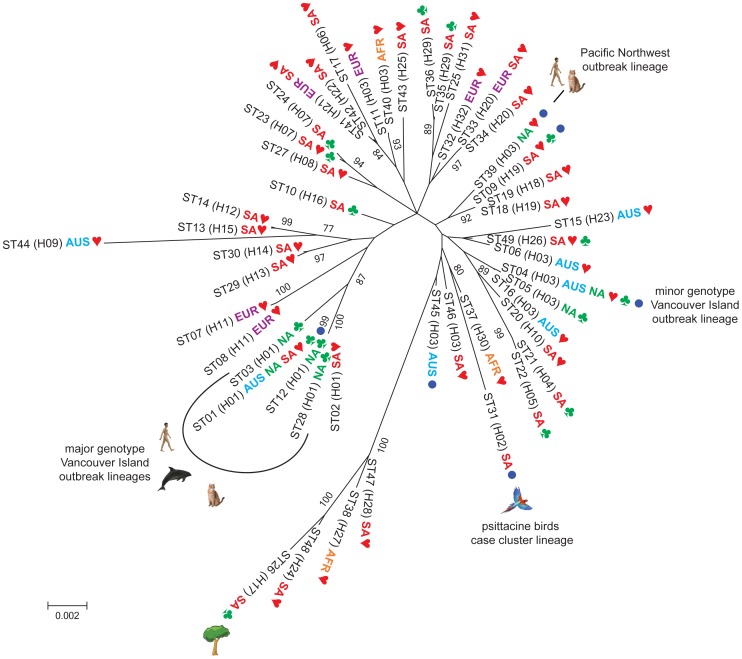
Unrooted Maximum Likelihood phylogenetic analysis of the concatenated SCAR-MLST dataset. The STs corresponding to haplotypes H17, H24, H27 and H28, being the ancestral lineages in the coalescence gene genealogy analysis, were found to be the most basal lineages when rooted with *C. gattii* AFLP4. Populations are indicated as AFR (Africa), AUS (Australasia), EUR (Europe), NA (North America) and SA (South America), followed by symbols indicating clinical (), environmental (♣) and/or veterinary (•) strains within the given ST. The most ancestral lineage H17 is indicated by a tree to highlight its environmental origin from a tree in pristine Amazon rainforest in Northern Brazil. STs that fell into H01 as the major genotype AFLP6A/VGIIa Vancouver Island outbreak lineage, H02 as the case cluster of *C. gattii* infections among psittacine birds in Brazil, H03 the minor genotype Vancouver Island outbreak lineage (ST4 and ST5), and the recently emerged genotype AFLP6C/VGIIc outbreak in the Pacific Northwest (ST39) are indicated with symbols referring to their respective origin (e.g. human referring to human infections, cat representing veterinary cases, dolphin representing infections among sea animals) (Fig. 1; Table 1 and S3). Bootstrap values are given for branches that are highly supported (≥75).

Summarized information for each of the 32 coalescence gene genealogy lineages, or haplotypes (A) (Fig. 1). (B) Represents SCAR-MLST sequence types (STs; Fig. 2) within the given haplotype with the number of representing strains between brackets, numbers between brackets are also indicating the source of these strains (clinical/environmental/veterinary). (C) Provides the number of strains per population, with AFR = Africa, AUS = Australasia, EUR = Europe, NA = North America and SA = South America. (D) Data is provided for those strains for which the macrophage J774 intracellular proliferation rate and BALB/c mice virulence assays were performed (Figs. 4 and 5), the strain numbers are given with between brackets their SCAR-MLST sequence type, the IPR values and ST_50_ mice survival with the number of mice per experiment provided between brackets.

**Table 2:** Genetic diversity of *C. gattii* populations based on AFLP, SCAR-MLST and microsatellite typing.

Population	Strains	differential AFLP			SCAR-MLST nuclear loci			Microsatellite typing			Combined datasets*			Allelic richness
		*n*_GENOTYPES_	*D*	*G_0_*	*n*_GENOTYPES_	*D*	*G_0_*	*n*_GENOTYPES_	*D*	*G_0_*	*n*_GENOTYPES_	*D*	*G_0_*	
**Africa**	10	5	0.822	5.56	3	0.689	2.63	6	0.844	4.17	9	0.978	7.14 (B)	2.95
**Australasia**	38	8	0.664	2.83	7	0.627	2.68	15	0.765	3.92	19	0.835	6.94 (BC)	3.42
**Europe**	6	5	0.933	4.46	6	1.000	5.44	5	0.933	4.46	6	1.000	7.00 (B)	3.73
**North America**	48	6	0.634	2.74	6	0.455	1.80	12	0.645	2.72	18	0.837	5.38 (C)	2.87
**South America**	76	33	0.877	7.44	31	0.870	7.06	37	0.929	12.03	48	0.951	36.10 (A)	5.85
**All**	178	52	0.928	13.14	49	0.907	10.23	72	0.940	15.55	97	0.969	37.74	N/A

Genetic diversity measured by Simpsons Diversity index (*D*) for each of the defined populations, as well as for all *C. gattii* AFLP6/VGII strains. Differential AFLP refers to the matrix of arbitrarily scored AFLP markers. Diversity of the SCAR-MLST has been given for all six loci as well as for each of the five nuclear loci. Diversity values range from 0.000 to 1.000. The higher the *D* value, the higher the genetic diversity is. Next to this, the Stoddart and Taylor genotypic diversity (*G*_0_) was calculated. This revealed a significant (*P*<0.05) higher genetic diversity in the South American *C. gattii* AFLP6/VGII population, the asterisks in the column ‘combined datasets’ indicates that the genotypic diversity of the populations followed by the same letters (between brackets) are not significantly different (*P*>0.05) based on pairwise bootstrap tests with 1000 permutations.

Generation time scaled coalescence analysis revealed that the major clade (H01–H16, H18–H23 and H25–H32) and the basal clade (H17 and H24) emerged approximately 17×10^6^ generations before present (gbp) (Fig. 1; Table 1). The lineage representing the major genotype involved in the Vancouver Island outbreak (H01) emerged approximately 1.4×10^6^ gbp. The estimated divergence time of the two closely related *C. gattii* strains A1M-R265 and CBS7750 (both lineage H01) was found to be approximately 51,449 years based on the number of synonymous nucleotide substitutions observed across 4,771 protein-coding orthologous genes.

The lineage representing the *C. gattii* case cluster among psittacine birds in Brazil (H02) emerged around 4.2×10^6^ gbp. A major diversification event that gave rise to a global lineage (H03) occurred approximately 5.3×10^6^ gbp and includes strains from the *C. gattii* outbreak in the Pacific Northwest and strains belonging to the minor genotype from the Vancouver Island outbreak. The latter were found to be genetically indistinguishable from strains obtained from a case cluster among over 100 sheep in Western Australia based on the consensus multi-locus sequence typing scheme for *C. neoformans* and *C. gattii* [4] (Fig. 1).

### Global Dispersal of *C. gattii* AFLP6/VGII

The coalescence gene genealogy analysis showed that the oldest lineages originated from Brazil. Approximately 12.4×10^6^ gbp a lineage dispersed to Africa, followed by a massive dispersal and recombination event approximately 5.3×10^6^ gbp that introduced *C. gattii* AFLP6/VGII into Australasia, Europe and North America (Fig. 1). Many lineages descended from this historical global dispersal event, which can be interpreted as a cascade of recombination and dispersal events rather than a single event. The most remarkable of these lineages (H03) was introduced into all inhabited continents and has resulted in recent outbreaks in North America and the case clusters in Australia and South America. Most of the lineages with a South American origin emerged during the past 3.4 – 4.0×10^6^ generations and were formed by a high rate of recombination events which seems to be the driving force behind the genetic diversification at this continent.

### An Independent Origin for the various Outbreaks

The genetic diversity of *C. gattii* genotype AFLP6/VGII was assessed by SCAR-MLST, microsatellite analysis and AFLP fingerprint analysis (Fig. 2, S1 and S2; Table 1 and S3). The Simpsons diversity index demonstrated that microsatellite typing had the highest discriminatory power (*D* = 0.940), followed by AFLP (*D* = 0.928) and SCAR-MLST (*D* = 0.909). Using the individual AFLP, SCAR-MLST and STR datasets the South American population had the highest genetic diversity, which was corroborated when these datasets were combined resulting in 58 genotypes among 76 strains (*D* = 0.951) (Table 2). The South American population was also found to have the highest genotypic diversity (*G*_0_) for each of the datasets, resulting in an overall significantly higher *G*_0_ of 36.10 (*P*<0.05) compared to the other four populations, supporting a South American origin of the global *C. gattii* AFLP6/VGII population (Table 2).

**Figure 3:**
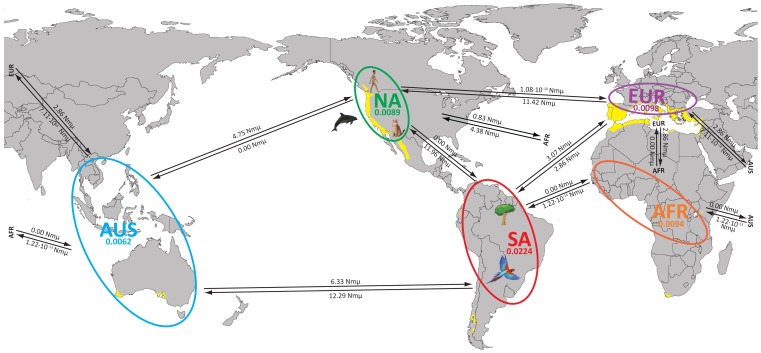
Population sizes and global migration patterns of *C. gattii*. The *C. gattii* AFLP6/VGII populations Africa (orange), Australasia (blue), Europe (purple), North America (green) and South America (red) are indicated, together with their mean population size (θ; Table S2), by similar coloured ellipses. Bi-directional historical mean migration rates are provided for each of the population combinations (Table S2). The approximate geographical location of the most basal lineage of coalescence gene genealogy (H17) is indicated with a tree in the Amazon. The outbreaks in North America are indicated with icons of human, cat and dolphin, and the parrot in the South American population indicates the case cluster of *C. gattii* infections among psittacine birds in Southern Brazil. Yellow coloured areas in the map represent localities with a Mediterranean climate according to the updated Köppen-Geigen classification (adapted from [37]).

As independent datasets, AFLP fingerprinting, SCAR-MLST, microsatellite analysis and re-analysis of published MLST data [3], [6] revealed that South America has the highest genetic diversity (Table 2). This is in agreement with previous investigations that showed the presence of all *C. gattii* genotypes in South America [17], [26], [27]. Mean population sizes and the migration patterns between population pairs were determined by using the full SCAR-MLST dataset. This analysis showed that the actual mean population size of South America (θ = 0.0224) is the largest, followed by Europe (θ = 0.0098), Africa (θ = 0.0094), North America (θ = 0.0089) and Australia (θ = 0.0062) (Fig. 3; Table S2). However, the lower and upper boundaries of the population size analysis showed a large variation, with the African and European populations being most variable. The North American *C. gattii* AFLP6/VGII population has a higher influx than efflux of migrants from the other populations with South America being the main contributor, followed by Europe, Australasia and Africa (Fig. 3; Table S2). The South American *C. gattii* AFLP6/VGII population has a higher efflux than influx of migrants to and from the other populations, with the exception of the African population that has an extremely low efflux of 1.22×10^−14^ Nmµ compared to no influx from South America (Fig. 3; Table S2). Efflux from the Australasian population is also higher to Africa, Europe and North America than *vice versa*, with South America as the exception since it contributes more to the influx into Australasia (Fig. 3; Table S2).

Maximum Likelihood phylogenetic analysis using the nuclear SCAR-MLST revealed that a highly supported basal lineage contained strains from Africa and South America (Fig. 2). All basal African strains were mating-type α, while both mating-types were present among the South American lineages. Other well supported lineages arose from this basal cluster, including three major clades with mating-type a strains that originate from South America, a mixed clade of Australian and North American mating-type α strains and a group of Vancouver Island outbreak mating-type α strains with a diverse basal group of African, Australasian, European and South American strains comprising different mating-types. The major Vancouver Island outbreak cluster contained three strains that originated from localities in Thailand (MC-S-239), Argentina (LA295) and Brazil (IFM47258) which were, except for MC-S-239, genetically indistinguishable from each other.

One hundred and thirty five isolates of *C. gattii* were used to re-analyze the updated ‘Fraser and Byrnes’ MLST dataset (original data taken from [3], [6]; Fig. S3), which revealed that several clades emerged from a basal South American cluster. Using this dataset, the large cluster of the major genotype *C. gattii* Vancouver Island outbreak strains (ST01) emerged from a diverse cluster containing Australasian (ST18, ST20), European (ST17) and South American (ST07, ST28, ST30) strains (Fig. S3).

### High Virulence is not Restricted to Outbreak Strains

**Figure 4:**
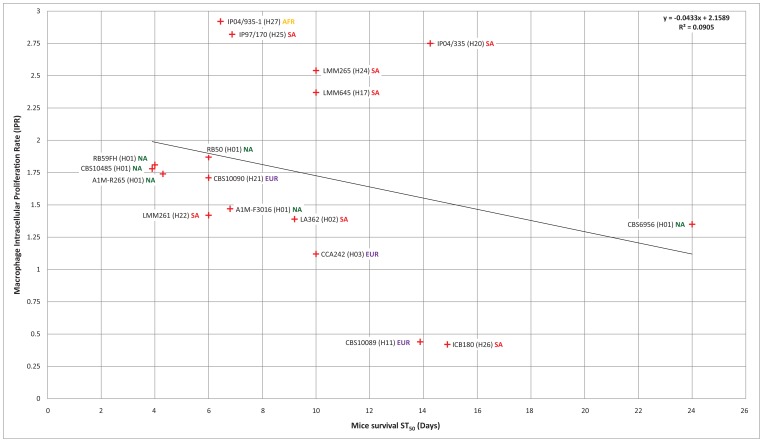
Pathogenicity based on macrophage J774 Intracellular Proliferation Rate (IPR) and survival of BALB/c mice. The fifty percent survival time (ST_50_) in days for BALB/c mice infected with *C. gattii* is presented on the x-axis and plotted against the Intracellular Proliferation Rate (IPR) of the same *C. gattii* strain inside murine J774 macrophage cells. Strains that were not virulent in the BALB/c mice experiment after 45 days are excluded from this graph (A1M-R272, IPR = 0.88; CBS1930, IPR = 1.14; CBS7750, IPR = 0.93; CBS8684, IPR = 0.90; and IHEM11489, IPR = 1.77). The origins of the strains are indicated as AFR (Africa), AUS (Australasia), EUR (Europe), NA (North America) and SA (South America). For detailed IPR and ST_50_ survival values see Table S3.

**Figure 5:**
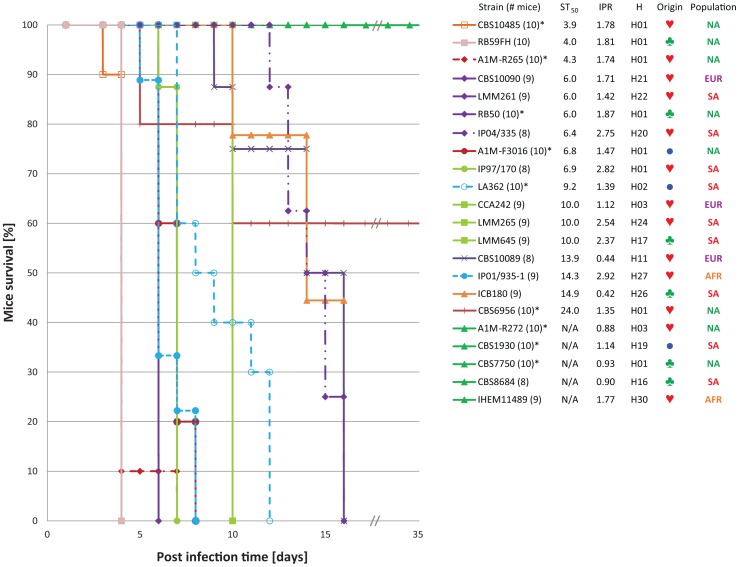
BALB/c mice survival curves. The Kaplan-Meier survival curves for each of the BALB/c mice virulence experiments carried out with 22 strains as listed from highest to lowest virulence potential in mice based on the ST_50_ values (Fig. 4; Table 1 and S3). Strains that showed identical progression of survival have similar curve profiles. The number of included mice per test are indicated between brackets, an asterisk after the strain number indicates that the data was from a previous mice virulence study [19]. Next to the strain number, the ST_50_, macrophage IPR and the coalescence gene genealogy haplotype number (H) are provided (Fig. 1; Table 1 and S3), followed by a symbol indicating a clinical (), environmental (♣) or veterinary (•) origin of the strain, as well as the population to which the strain belongs (AFR = Africa; AUS = Australasia; EUR = Europe; NA = North America; SA = South America). Note that strains A1M-R272, CBS1930, CBS7750, CBS8684 and IHEM11489 are non-virulent until day 45.

Previous studies indicated that *C. gattii* AFLP6A/VGIIa, the so-called major Vancouver Island outbreak genotype, and strains with genotype AFLP6C/VGIIc from the Pacific Northwest outbreak are highly virulent in different host models, while strains that belong to the minor Vancouver Island outbreak genotype AFLP6B/VGIIb were less virulent or non-virulent [3], [6], [18], [19]. To further elucidate the virulence potential among strains within the basal lineages of the coalescence tree, we investigated the virulence of 22 *C. gattii* strains using mice and macrophage pathogenicity assays ([3], [19]; Figs. 1, 4 and 5; Table S3). This revealed that the ancestral lineages in the coalescence analysis represented by the South American strains LMM645 (H17; mating-type a), isolated from a tree in a pristine Amazon rainforest, and the clinical strain LMM265 (H24; mating-type α) were more virulent (ST_50_ = 10.0±0.0 days each) when compared to other strains (Figs. 4 and 5). Despite the fact these strains show a dramatic virulent potential to cause disease in the BALB/c mice model, these two South American strains were significantly (*P*<0.001) less virulent than strains with the major genotype AFLP6A/VGIIa from the Vancouver Island outbreak region, which had ST_50_ values ranging from 3.9±0.1 to 6.8±0.3 days (Figs. 4 and 5; Table S3). The macrophage intracellular proliferation rates (IPR) of these two South American strains were among the highest observed with values of 2.54 for LMM265 and 2.37 for LMM645 ([3], [19]; Table S3). The highest IPR values were observed for the three mating-type α strains IP2001/935-1 (H27; Africa), IP1997/170 (H25; South America) and IP2004/335 (H20; South America) which had IPR values of 2.92, 2.82 and 2.75, respectively. These high IPR values were also reflected by high pathogenicity in BALB/c mice (Fig. 4; Table S3), although we occasionally noted that a high IPR value does not necessarily predict a high virulent phenotype in the BALB/c mice model (Fig. 4; Table S3). Five strains had a non-virulent phenotype when tested in the BALB/c mice model, which could not always be predicted based on the macrophage intracellular proliferation assay as shown by strain IHEM11489 (IPR = 1.77; H30, Africa). The mice and macrophage model were in agreement for the non-virulent strains A1M-R272 (IPR = 0.88; H03, Vancouver Island outbreak), CBS7750 (IPR = 0.93; H01. Vancouver Island outbreak) and CBS8684 (IPR = 0.90; H16, South America) (Table S3).

### Recombination is High among South American Strains

The presence of both mating-types in a nearly equal rate (mating-type **a** : α = 0.8∶ 1) in the South American population suggests that recombination events are likely to occur at this locality. The majority of these mating-type **a** strains sampled from various localities in Colombia were observed to be clonal. *STE12* mating-type analysis of the *C. gattii* AFLP6/VGII strains confirmed that the population in Mediterranean Europe contained mating-type **a** and α strains as well, while the African, Australasian and North American populations were mating-type α only (Table S3).

Ancestral recombination graph analysis of SCAR-MLST sequences showed the presence of extensive successive recombination events in the history of the current global population of *C. gattii* AFLP6/VGII, as illustrated by the presence of 167 historical recombination events (Fig. S4). The level of recombination detected by the ancestral recombination graph analysis differed widely among populations. The number of historical recombination events, based on ancestral recombination graph analysis, was disproportionally high in South America (110 events), whereas this value was low in the Australasian, European and North American populations (8, 5 and 1 historical recombination events, respectively) and undetectable in the African population. Estimates of the minimum number of recombination events (*R*_M_) in the current population of *C. gattii* AFLP6/VGII corroborated the ancestral recombination graph analysis results, indicating a high number of recombination events (*R*_M_ = 22) in South America versus those in the other populations (*R*_M_ = 4 for Australasia; *R*_M_ = 2 for Europe; *R*_M_ = 0 for Africa and North America) (Table S4). The Φ_W_ test provided further evidence for actual signs of recombination in the SCAR-MLST dataset (*P*<0.001) but not within the extended ‘Fraser and Byrnes’ MLST dataset (*P* = 0.196). When the SCAR-MLST data was split into the five populations, evidence for recombination was found by the Φ_W_ test in the Australian (*P* = 0.033), European (*P* = 0.044), North American (*P*<0.001) and South American (*P*<0.001) populations. CASS network analysis also revealed highly supported signs of actual recombination in the SCAR-MLST dataset (Fig. S5). Thus, coalescence gene genealogy, ancestral recombination graph and phylogenetic approaches indicate that two large clades, including the Vancouver Island and Pacific Northwest outbreak lineages (H01 and H03, respectively) and a large cluster of strains from other geographical localities, were a result of ancient recombination events between ancestors of the current, but basally positioned, South American lineages and either the South American lineage H07 or one or more currently unknown ancestor(s) of the recombinant strain ENV133 (H01; Vancouver Island outbreak) (Fig. S5).

## Discussion

Our study demonstrates that the current global *C. gattii* AFLP6/VGII population has most likely a South American origin rather than being introduced from another geographical locality such as Australia, Africa or Europe, as was proposed in previous studies [3], [6]. The presence of *C. gattii* AFLP6/VGII in the natural and pristine environment of the Amazon rainforest [21] makes it highly unlikely that the fungus was introduced from Australia into South America via import of Australian *Eucalyptus* seeds containing *C. gattii*, as suggested [3], [6] and disputed [22] before. Therefore an ‘Out of the Amazon rainforest’ model is the most plausible scenario for the origin of the AFLP6/VGII outbreaks that emerged in British Columbia (Canada) and the Pacific Northwest (USA), as well as the case clusters among sheep in the Perth region (Australia) and psittacine birds in Brazil.

Migration patterns between the *C. gattii* AFLP6/VGII populations pairs showed that South America is the major donor of migrants for the other populations, with Africa being the exception due to a very small influx of genetic material into South America, while this populations seems not to contribute to the genetic influx into the African population (Fig. 3; Table S2). The mean South American population size was found to be the largest, distantly followed by the mean population sizes of Europe, Africa, North America and Australia, respectively. However, this needs to be interpreted cautiously because the variation in the lower and upper boundaries for the population size measurements were relatively large, especially for the populations of Africa and Europe that contained the smallest set of strains (Table S2). Taken together, the population size and migration analyses are pointing towards a South American origin of the global *C. gattii* AFLP6/VGII population.

Phylogenetic analyses demonstrated that the recently emerged Pacific Northwest outbreak lineage is only distantly related to the major Vancouver Island outbreak genotype AFLP6A/VGIIA, which is in agreement with previous studies 3,28. The SCAR-MLST phylogenetic (Fig. 2), AFLP and microsatellite analyses (Fig. S1 and S2) showed that the Pacific Northwest outbreak strains are genetically not closely related to those from the Vancouver Island outbreak, but that they clustered together with strains from South America (Figs. 2, S1, S2, S3). This indicates that the genetic origin of the Pacific Northwest *C. gattii* outbreak strains is most likely in South America, and this seems also to be the case for the Pacific Northwest lineage that apparently emerged from recombination events with one or more South American historical lineages as depicted by our phylogenetic and recombination network analyses (Fig. 2, S4 and S5). Notably, the investigated population of South American strains were found to have a nearly equal rate of mating-type **a** and α (0.8∶ 1), as opposite to the other populations were mating-type **a** strains were absent or outnumbered by mating-type α strains, an indication that regular mating events play a more important role in the South American population than so-called same-sex mating [6]. The implication of this observation is that the Pacific Northwest outbreak lineage was introduced into North America from South America independently from the Vancouver Island outbreak lineages. Similarly, the *C. gattii* AFLP6/VGII case cluster among psittacine birds in Brazil is genetically not closely related to any of the other outbreaks (Fig. 2). In contrast, based on the data obtained using a consensus MLST scheme for *C. neoformans* and *C. gattii* the isolates from the *C. gattii* AFLP6/VGII case cluster among sheep in Western Australia were recently found to be genetically indistinguishable from strains with the minor genotype that also occur on Vancouver Island (Figs. 2, S1 and S2) [4].

Contrary to previous assumptions [3], [6], [18], [19], the virulence trait is not restricted to Vancouver Island outbreak strains that belong to the major genotype AFLP6A/VGIIA, virulent and non-virulent phenotypes co-occur within recently emerged *C. gattii* AFLP6/VGII lineages (Fig. 1). *Cryptococcus gattii* strains from the Pacific Northwest outbreak had a similar virulence potential in mice and macrophage pathogenicity assays as strains from British Columbia (this study; [*3*]).

The mechanism for the ancient global spread remains as yet unclear. Historical and actual recombination events shaped the genetic armamentarium of *C. gattii* AFLP6/VGII making it likely that the fungus became favorably adapted to ecological zones where the species has not been reported before [3]. Long distance dispersal may have contributed to the intercontinental movement of *C. gattii*. Unusual, extreme weather events, such as cyclonic winds, may have a great effect on the dispersal of microbial organisms [29], [30]. Aerial movements of human pathogenic fungi have not been investigated at a large scale, but it has been shown that fungi, including *Cryptococcus* species, are present in samples from dust storms, which might be a possible mechanism of dispersal for global spread of *C. gattii* [30]. Moreover, sea- and freshwater movements may also have played a role in the dispersion. *Cryptococcus gattii* AFLP6/VGII yeast cells are able to survive in water with the same salinity as seawater, and the AFLP6/VGII genotype has been found to occur in seawater and marine mammals are regularly infected by this yeast [7], [25], [31].

During the past two decades several *C. gattii* AFLP6/VGII outbreaks have been observed in British Columbia (Canada), the Pacific Northwest (USA), case clusters among psittacine birds in Southern Brazil, an epidemic among sheep in Western Australia, and a higher prevalence of infections among immunocompetent native children in Pará state, North East Brazil [3], [4], [6]–[8], [12], [13]. During the same period, another genotype of *C. gattii* (AFLP4/VGI) emerged in the European Mediterranean area where it caused several small outbreaks among goats [32]. Autochthonous *C. gattii* AFLP6/VGII infections were observed in Greece, and a recent survey revealed that *C. gattii* AFLP4/VGI occurs widespread in indigenous tree species in the Spanish Mediterranean region [16], [33]–[35]. These examples indicate that *C. gattii* is moving from its endemic tropical and subtropical regions towards more temperate climate zones, such as the European Mediterranean region [34], [35]. As such the hypothesis of a sudden emergence of a single virulent lineage that may have given rise to the various North American outbreaks can be ruled out as all the strains involved in the various outbreaks and case clusters appear to have a different genetic composition.

Most Vancouver Island outbreak *C. gattii* strains with the major genotype AFLP6A/VGIIa are highly virulent in two host models, irrespective of their clinical, veterinary or environmental source (this study; [17]). The clinical strain CBS6956 (H01), however, showed an attenuated virulence [6], [19] and was isolated from sputum of a patient in Seattle in the 1970s. This strain was isolated from a patient approximately thirty years prior to the onset of the current Canadian and USA outbreaks [7]. This observation suggests that the genotype occurred in the outbreak region decades before the emergence of the outbreak. Moreover, a recent serological study revealed that approximately 65% of the residents of Vancouver Island, and 18% of the miners sampled between 1982–1984, had antibodies against *C. gattii* [36]. Taken together, these observations suggest that the outbreak lineage was present in the region at least several decades before the emergence of the Canadian outbreak, but that it took until the end of the 20^st^ century before the outbreaks emerged.

According to the recently updated Köppen-Geigen climate classification system [37] the outbreak and case cluster localities in Western Australia, Mediterranean Europe and North America are all situated in regions with a Mediterranean sea climate characterized by either dry summers or dry winters with an average temperature of 22°C during the warmest month. It is possible that recent changes in environmental conditions [38] favored the dispersal of the yeast into regions with more temperate climate zones and, thus, contributed to the development of the various outbreaks. The notion that several *C. gattii* infections co-occurred in time in Mediterranean climate zones in Europe, Australia and North America, supports this latter supposition (this study; [3], [4], [7], [14], [33]–[35], [37]. Estimates of the climate change velocity indicated that temperate and Mediterranean climate zones belong to the group with the highest velocity in temperature change [39]. Thus an increase in the average annual temperature might influence the emergence and severity of pathogenic microorganisms in temperate climate zones [40]. The increase of global temperatures and factors that relate to this, such as precipitation, humidity, air and water temperature, has changed the distribution patterns of a variety of pathogenic microorganisms affecting plants and animals [40], [41] and it seems plausible that this holds true for the recent range expansion of *C. gattii* AFLP6/VGII. However, the higher local prevalence among children in Northern South America and the case cluster of *C. gattii* AFLP6/VGII among psittacine birds in Brazil is a further indication that this yeast originated from this region thus reflecting its endemicity. Probably, these infections relate to yet unknown niche disturbances of the pathogens that may be related to changed landscape use, which needs further assessment.

In conclusion, coalescence gene genealogy and phylogenetic analyses showed that the recent emergence of *C. gattii* AFLP6/VGII into temperate Mediterranean climate regions was most likely due to ancient dispersal out of the tropical Amazon rainforest. The virulent trait is not restricted to the outbreak lineages from Canada (major genotype AFLP6A/VGIIa) and the Pacific Northwest (genotype AFLP6C/VGIIc), but is present among all globally sampled coalescence gene genealogy lineages, including the most basal lineages from South America. Non-virulent strains co-occurred with virulent ones in the Vancouver Island outbreak lineage H01 (Fig. 1). Historical recombination events occurred in high rates within the South American population and are likely to be the driving force behind the diversification of the pathogen and the erratic phylogenetic presence of virulence among the lineages. We hypothesize that ecological change, such as alterations in global climate patterns, allowed *C. gattii* to emerge in temperate Mediterranean climate zones at various places on Earth, where apparently ideal, but yet unknown, conditions have led to population expansion and thus disease outbreaks in a diverse range of hosts.

## Materials and Methods

### Ethics Statement

Procedures, care and treatment of the mice were carried out according to the principles of humane treatment outlined by the Guide for the Care and Use of Laboratory Animals of the Hebrew University, approved by the Committee for Ethical Conduct in the Care and Use of Laboratory Animals (approval number OPRR-A01–5011) and in full compliance with the Israel animal welfare act (law 5754–1994 and 5761–2001). The study was approved by the joint ethics committee for animal welfare (IACUC) of the Hebrew University and the Hadassah Medical Centre (Jerusalem). The facilities of the Hebrew University are accredited by the Association for Assessment and Accreditation of Laboratory Animal Care International (accreditation number 1285), experiments were performed in full compliance with national and international guidelines.

### Strains and media

A global set of 178 *C. gattii* strains was selected based on previous PCR fingerprinting and AFLP genotype analyses and included strains involved in the Vancouver Island outbreak (major and minor outbreak lineages AFLP6A/VGIIa and AFLP6B/VGIIB, respectively), the Pacific Northwest outbreak AFLP6C/VGIIc, the case cluster among psittacine birds in Brazil, strains from Western Australian, and strains obtained from pediatric cryptococcosis cases (this study; [3], [4], [7], [8], [13], [16], [21], [26], [27]. The strains came from Africa (*n* = 10), Australasia (*n* = 38), Europe (*n* = 6), North America (*n* = 48) and South America (*n* = 76) (Table S3). Eleven strains were used as reference strains for molecular studies: WM148 ( = CBS10085; αA; AFLP1/VNI), WM626Brown ( = CBS10083; αA; AFLP1A/VNII), WM626White ( = CBS10084; αA; AFLP1A/VNII), WM629 (CBS10079; αD; AFLP2/VNIV), WM628 (CBS10080; αAaD; AFLP3/VNIII), WM179 (CBS10078; αB; AFLP4/VGI), WM161 (CBS10081; αB; AFLP5/VGIII), WM779 ( = CBS10101; αC; AFLP7/VGIV), WM276 ( = CBS10510; αB; AFLP4/VGI), A1M-R265 ( = CBS10514; αB; AFLP6A/VGIIa) and A1M-R272 ( = CBS10865; αB; AFLP6B/VGIIb) [16]. Strains were cultured and maintained on YPGA-medium (1% w/v yeast-extract, 1% w/v peptone, 2% d-glucose and 2% w/v agar) at 25°C and stored at 4°C on slants or in glycerol stocks at −196°C in liquid nitrogen.

### Intracellular Macrophage Proliferation Assay (IPR)

A subset of *C. gattii* AFLP6/VGII strains, indicated in Fig. 4 and Table S3, was tested for their ability to proliferate in J774 macrophages as a measurement of their virulence potential, as described previously by Ma et al. [19]. In brief, cryptococcal cells were opsonized with 18B7 antibody (a kind gift of Arturo Casadevall) and then exposed to J774 macrophage cells for two hours. Each well was then repeatedly washed with phosphate-buffered saline (PBS) to remove extracellular yeast cells before 1 ml of fresh serum-free DMEM was added. At time points of 0 hours (immediately after infection), 18 hours and 24 hours, media was removed and replaced with 200 µl of sterile dH_2_O to lyse macrophage cells. After 30 minutes, the intracellular yeast were released and collected and another 200 µl dH_2_O was added to collect the remaining yeast cells. Intracellular yeast were then mixed with Trypan Blue at a 1∶1 ratio and the live yeast cells were counted. The IPR value was calculated by dividing the maximum intracellular yeast number (either at 18 or 24 hours) by the initial intracellular yeast number at T = 0. Each assay was repeated at least three times on different occasions.

### Mice Virulence Study

Virulence of the *C. gattii* strains was studied in a BALB/c mice model as described by Ma et al. [19]. Briefly, a single bolus injection of a 0.2 ml yeast suspension (2–5×10^6^ yeast cells from a 48 h culture on Sabouraud Dextrose agar at 30°C) in PBS were injected into the tail veins of male albino BALB/c mice (body weight of 20–23 g). The tail vein injection method was preferred above the use of the inhalation model since the former method provides a more controlled measurement of the number of yeast cells entering the blood system compared to the inhalation model. A hemocytometer was used to determine the concentration of yeast cells, while the viable count was measured as the number of CFU on Sabouraud Dextrose agar plates after 24–48 h of incubation at 30°C. For each of the tested *C. gattii* strains, a set of 8–10 mice were included, based on IPR outcome of the inoculated yeast strain, that were maintained in separate cages. The genome sequenced reference strain A1M-R265 was used as a control for each experiment. The survival rate of the mice was recorded daily, for up to 45 days, in such a way that the animal handlers were unaware of the corresponding IPR values for the strains tested. Statistical analysis was performed using the Kaplan-Meier method in SPSS v21 (SPSS, Chicago, IL).

### Ploidy Analysis and Generation Time Determination

Strains were cultivated for 48 h on malt-extract agar at 25°C. The carbon source *myo*-inositol, typically assimilated by tremellaceous yeasts, was chosen for the generation experiment since this is an important factor for the sexual development and virulence potential of *Cryptococcus*, and it is abundantly present in the human central nervous system [42]. The complete procedure is described in Material and Methods S1 and includes Figure S6.

The ploidy of selected *C. gattii* strains (Table S3) was analyzed according to the method described by Bovers et al. [15]. The two haploid reference strains A1M-R265 and A1M-R272 and the homozygote diploid *C. gattii* strain RB59JF were included [6]. Further details are provided in Material and Methods S1.

### DNA Extraction, Mating-type Determination, various Genotyping Approaches

Genomic DNA was extracted as described in Material and Methods S1Material and Methods S1. All *C. gattii* strains were subjected to mating-type determination by partial amplification of the *STE12* gene, as described previously [16], and was performed to determine mating-type a and α specific regions of all strains using *C. gattii* strains CBS1930 (aB) and CBS6956 (αB) as a reference. Strains were genotyped using various approaches as described in Material and Methods S1, and include amplified fragment length polymorphism (AFLP) analysis, an approach called sequence characterized AFLP regions multi-locus sequence typing (SCAR-MLST) that was developed based on sequenced polymorphic markers obtained by differential AFLP fingerprinting analysis [43], a set of strains (Table S3) were used to supplement the MLST dataset of Byrnes et al. [3] and Fraser et al. [6] and, finally, a novel microsatellite typing scheme was established consisting of ten markers. A detailed workflow for each of the molecular techniques is described in Material and Methods S1 and includes Table S5.

### DNA-based Analyses

SCAR-MLST and ‘Fraser & Byrnes’ MLST datasets were subjected to various sequence based analyses that include a search for the best fitting nucleotide substitution model, bootstrapped Maximum Likelihood phylogenetic analysis, genetic diversity analyses, recombination analyses, ancestral recombination graph analysis and coalescence gene genealogy analysis. The detailed workflow for these analyses is described in Material and Methods S1 and includes Table S5.

### Whole Genome Sequencing

To estimate the divergence time of two closely related *C. gattii* AFLP6/VGII strains the environmental and non-virulent *C. gattii* strain CBS7750 and the virulent clinical reference strain A1M-R265 were subjected to whole genome sequencing with an approximately 350 fold coverage of the genomes. The workflow that consists of the MIRA mapping assembly, de novo assembly using SOAP, SNP and indel detection using Varscan and the genome-wide divergence time estimate is provided in Material and Methods S1.

## Supporting Information

Figure S1 **Microsatellite analysis of** ***C. gattii*** **AFLP6/VGII strains.** Minimum spanning tree analysis based on ten microsatellite loci showing the genetic diversity of the complete set of *C. gattii* AFLP6/VGII strains and especially that of the South American population (red). Case cluster and outbreak related clusters are indicated. Colours represents the populations of Africa (yellow), Australasia (blue), Europe (purple), North America (green) and South America (red). Circle sizes are relative to the number of strains that are represented by that microsatellite type. Connecting lines between circles indicate the number of different microsatellite between both groups. Thick black lines represent one different locus, thin black two loci, dashed black line three loci, dashed dark grey line four loci and five or more different loci are indicated with dashed light gray lines plus number of different loci.
(TIF)

Figure S2 **Neighbour Joining analysis of arbitrarily scored AFLP markers.** The matrix of absent (0) and present (1) differential AFLP markers (Table S3) has been used to generate a Neighbour Joining tree including an outgroup of *C. neoformans* and *C. gattii* reference strains. Haplotype numbers are provided as shown in the coalescence gene genealogy and populations are indicated behind the strain numbers as AFR (Africa), AUS (Australasia), EUR (Europe), NA (North America) and SA (South America).
(TIF)

Figure S3 **Phylogenetic analysis of the extended ‘Fraser & Byrnes’ MLST dataset.** Unrooted Maximum Likelihood phylogenetic analysis of the sequence types found within the concatenated dataset of seven nuclear MLST loci as provided by Byrnes et al. [S14] and Fraser et al. [S4] and extended with additional strains in the current study. The most ancestral lineage (here ST07) from the SCAR-MLST based coalescence gene genealogy analysis is indicated in this figure with a tree to highlight its origin from pristine Amazon rainforest. When rooted with *C. gattii* genotype AFLP4/VGI, AFLP5/VGIII and AFLP7/VGIV, strains within ST18 are basal to the other lineages. The outbreaks in North America are indicated with icons of human, cat and dolphin, and the parrot in the South American population highlight the case cluster of *C. gattii* infections among psittacine birds in Southern Brazil. Populations are indicated behind strain and haplotype numbers as AFR (Africa), AUS (Australasia), EUR (Europe), NA (North America) and SA (South America).
(TIF)

Figure S4 **Ancestral recombination graph analysis based on SCAR-MLST.** The ancestral recombination graph shows historical recombination events that gave rise to the current population structure represented by the clone corrected 49 sequence types (STs) observed within the concatenated nuclear SCAR-MLST dataset (Table S3). The ST01/ST02 lineage represents strains with the major Vancouver Island outbreak genotype AFLP6A/VGIIa (indicated with a human, cat and dolphin symbol to represent the clinical and veterinary strains), similarly indicated is ST04 representing the minor genotype AFLP6B/VGIIb. The recently emerged *C. gattii* AFLP6C/VGIIc Pacific Northwest outbreak (ST39) has been indicated with a human and cat symbol. The tree icon indicates the most ancestral lineage that was isolated from a tree in the pristine Amazon rainforest (ST26). The 167 blue circles indicate historical recombination breakpoint positions within the concatenated nuclear SCAR-MLST dataset.
(TIF)

Figure S5 **CASS network analysis based on SCAR-MLST.** Recombination network analysis based on the CASS algorithm shows that several recombination events occurred in the current global population and that the majority of parental donors came from the South American population. Populations are indicated behind strain and haplotype numbers as AFR (Africa), AUS (Australasia), EUR (Europe), NA (North America) and SA (South America). The outbreaks in North America are indicated with icons of human, cat and dolphin, and the parrot-icon in the South American population highlight the case cluster of *C. gattii* infections among psittacine birds in Southern Brazil.
(TIF)

Figure S6 **Growth curves of** ***C. gattii*** **AFLP6/VGII strains on** ***myo*** **-inositol.** Growth curves of the three replicated growth rate experiments for A1M-R265 (major genotype AFLP6A/VGIIa Vancouver Island outbreak lineage) and CBS1930 (Aruba). The formula for each of the trendlines is provided as this is part of the formula to calculate the generation time. For the coalescence gene genealogy generation time scale calculation, the average value is used from all six growth rate experiments.
(TIF)

Table S1 **Map of informative sites used for the coalescence gene genealogy analysis.** The clone corrected SCAR-MLST data has been collapsed into haplotypes after removal of homoplasious sites. Informative sites within the complete dataset, and among the 32 haplotypes identified, are provided per nuclear SCAR-MLST locus that has been provided as the Fragment-number. IGS1 refers to the Intergenic Spacer 1 region (see Table S5). Colours used for each of the loci correspond with those used to mark the mutation events along the coalescence gene genealogy in Fig. 1.
(PDF)

Table S2 **Population sizes and historical migration rate estimates among** ***Cryptococcus gattii*** **populations.** The population sizes and migration rates were estimated using Migrate v2.3 (http://popgen.scs.fsu.edu) based on the five nuclear SCAR-MLST loci. The centre column shows the mean Nmµ value, while the left flanking values represents the lower (left value) and upper (right value) 95% confidence interval values.
(PDF)

Table S3 **List of strains and background information.**
(XLSX)

Table S4 **Overview of molecular variation, including number of haplotypes, nucleotide diversity, estimates of theta based on the number of segregating sites and recombination parameters.** The genetic diversity is provided per population and for all strains, as well as for each locus independently and the mean value for the given values. Given values are for the number of sites within an alignment with or without alignment gaps, the nucleotide diversity per site (π) corresponding to the average number of nucleotide differences per site between two sequences, the number of segregating or polymorphic sites (S), the Watterson’s estimated θ per locus and per nucleotide, the ‘Hud4Nc per site’ value representing the recombination rate per generation between the most distant nucleotides. The last two values listed the number of recombination events based on the ‘four gametic test’ and the minimum number of recombination events in the history of the sample (Note that provides an underestimation of the number of recombination events).
(PDF)

Table S5 **Primers used for SCAR-MLST and microsatellite typing.**
(PDF)

Materials and Methods S1 **Supporting Information.**
(DOCX)
