# Supplementary figures and images for: Ancient Dispersal of the Human Fungal Pathogen Cryptococcus gattii from the Amazon Rainforest
Source: PLoS One. 2013 Aug 7;8(8):e71148. doi: 10.1371/journal.pone.0071148 (PMC3737135; doi:10.1371/journal.pone.0071148)

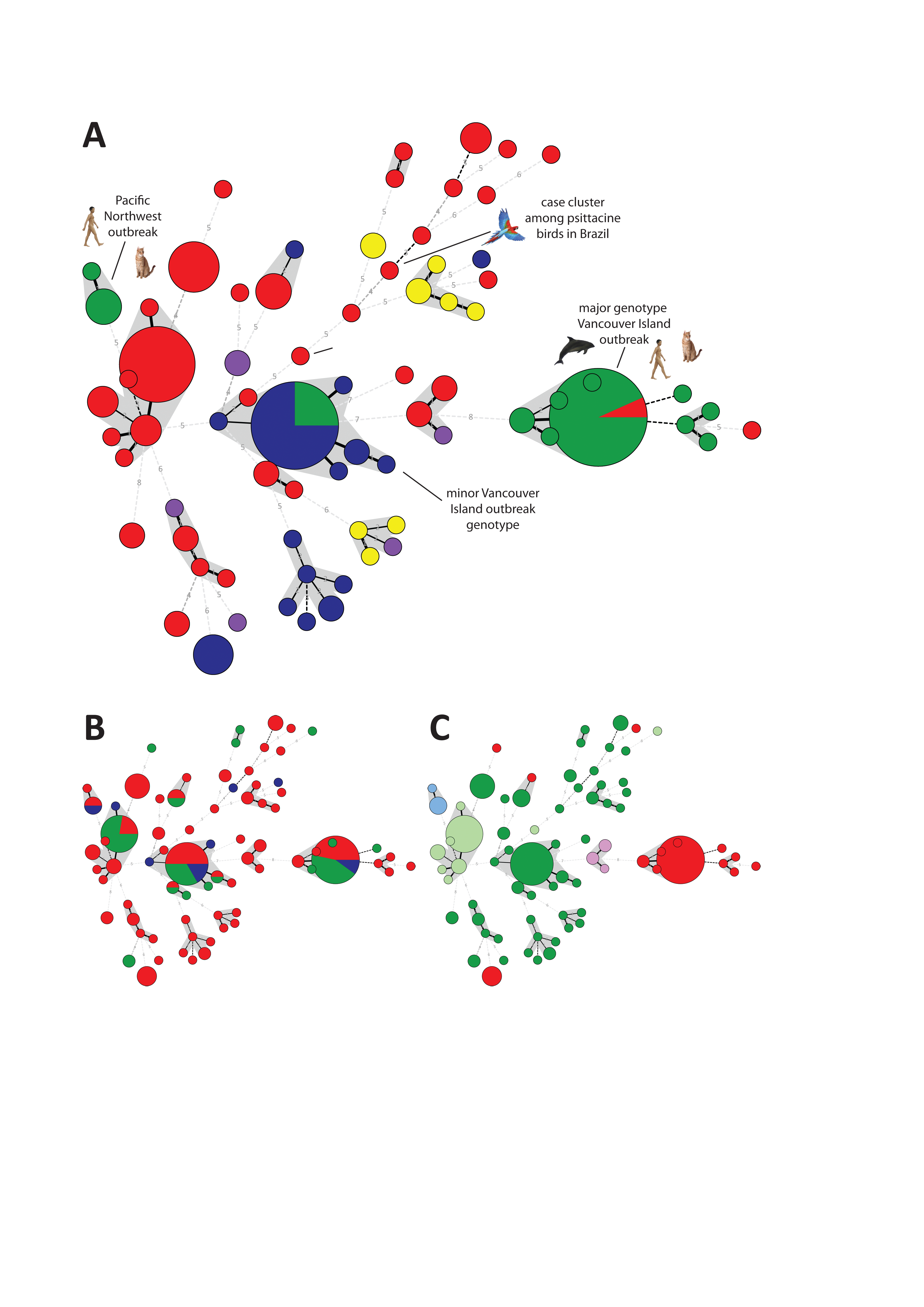

Supplement: Figure S1 — Microsatellite analysis of C. gattii AFLP6/VGII strains. Minimum spanning tree analysis based on ten microsatellite loci showing the genetic diversity of the complete set of C. gattii AFLP6/VGII strains and especially that of the South American population (red). Case cluster and outbreak related clusters are indicated. Colours represents the populations of Africa (yellow), Australasia (blue), Europe (purple), North America (green) and South America (red). Circle sizes are relative to the number of strains that are represented by that microsatellite type. Connecting lines between circles indicate the number of different microsatellite between both groups. Thick black lines represent one different locus, thin black two loci, dashed black line three loci, dashed dark grey line four loci and five or more different loci are indicated with dashed light gray lines plus number of different loci. (TIF) [file pone.0071148.s001.tif]

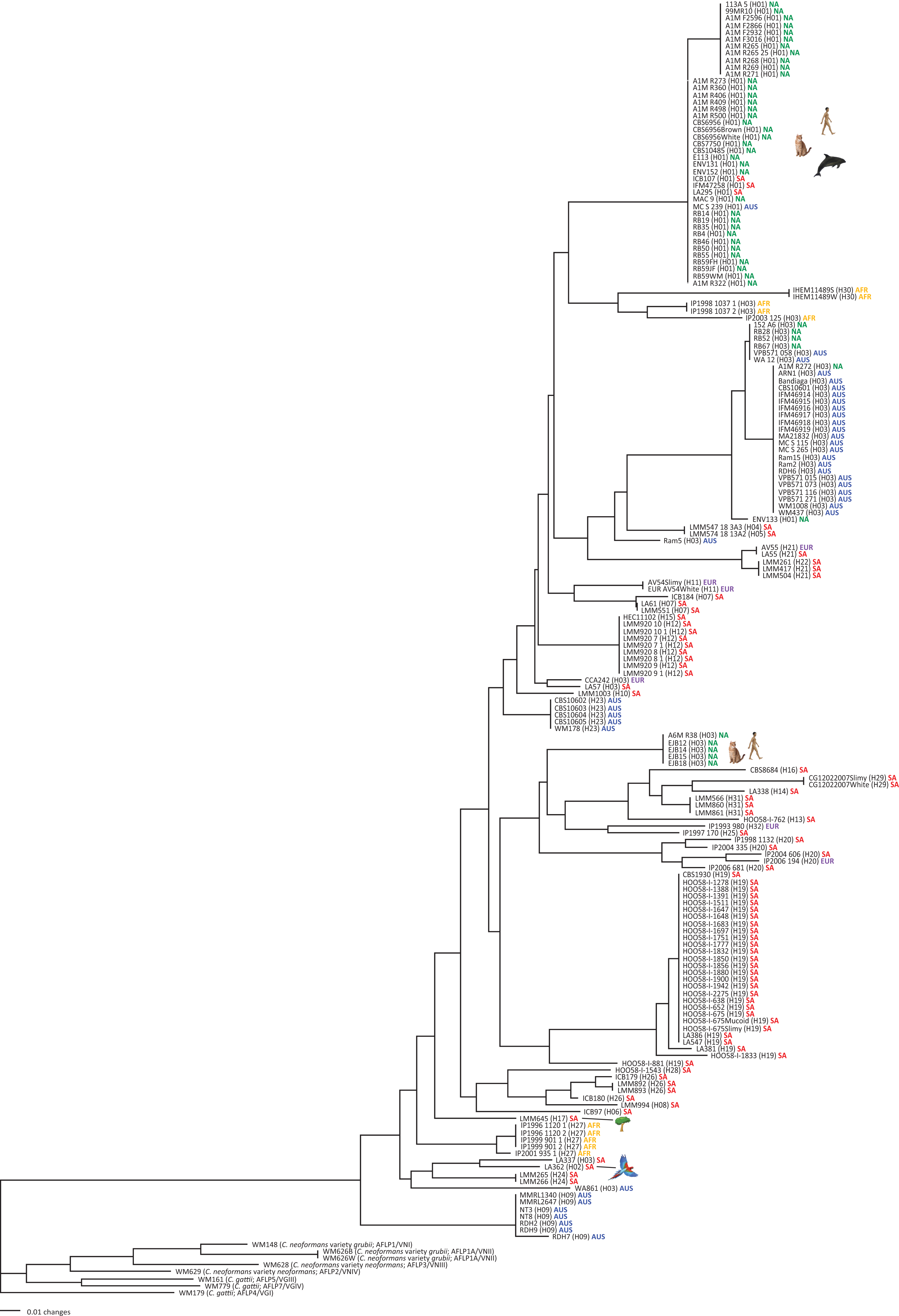

Supplement: Figure S2 — Neighbour Joining analysis of arbitrarily scored AFLP markers. The matrix of absent (0) and present (1) differential AFLP markers (Table S3) has been used to generate a Neighbour Joining tree including an outgroup of C. neoformans and C. gattii reference strains. Haplotype numbers are provided as shown in the coalescence gene genealogy and populations are indicated behind the strain numbers as AFR (Africa), AUS (Australasia), EUR (Europe), NA (North America) and SA (South America). (TIF) [file pone.0071148.s002.tif]

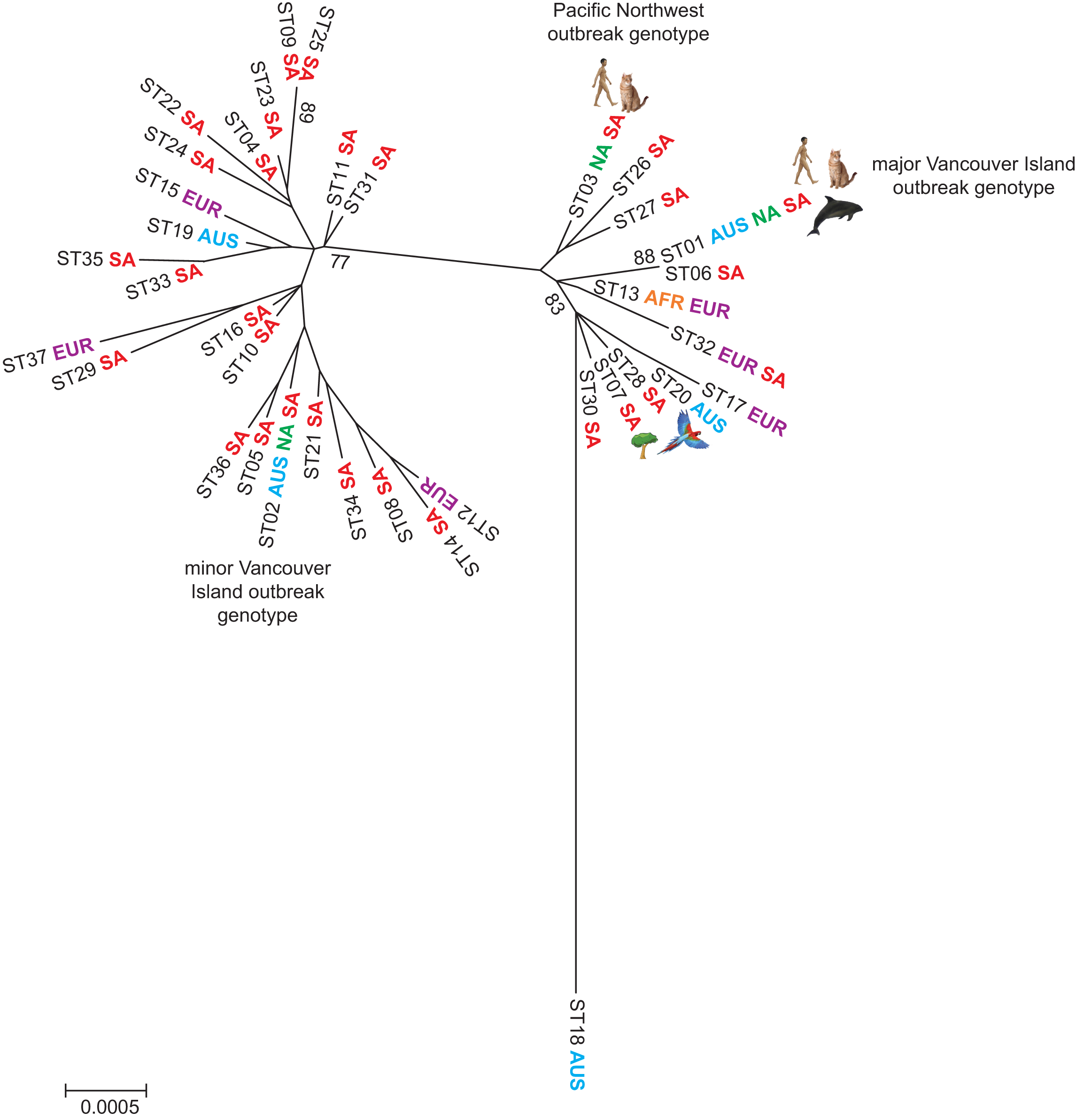

Supplement: Figure S3 — Phylogenetic analysis of the extended ‘Fraser & Byrnes’ MLST dataset. Unrooted Maximum Likelihood phylogenetic analysis of the sequence types found within the concatenated dataset of seven nuclear MLST loci as provided by Byrnes et al. [S14] and Fraser et al. [S4] and extended with additional strains in the current study. The most ancestral lineage (here ST07) from the SCAR-MLST based coalescence gene genealogy analysis is indicated in this figure with a tree to highlight its origin from pristine Amazon rainforest. When rooted with C. gattii genotype AFLP4/VGI, AFLP5/VGIII and AFLP7/VGIV, strains within ST18 are basal to the other lineages. The outbreaks in North America are indicated with icons of human, cat and dolphin, and the parrot in the South American population highlight the case cluster of C. gattii infections among psittacine birds in Southern Brazil. Populations are indicated behind strain and haplotype numbers as AFR (Africa), AUS (Australasia), EUR (Europe), NA (North America) and SA (South America). (TIF) [file pone.0071148.s003.tif]

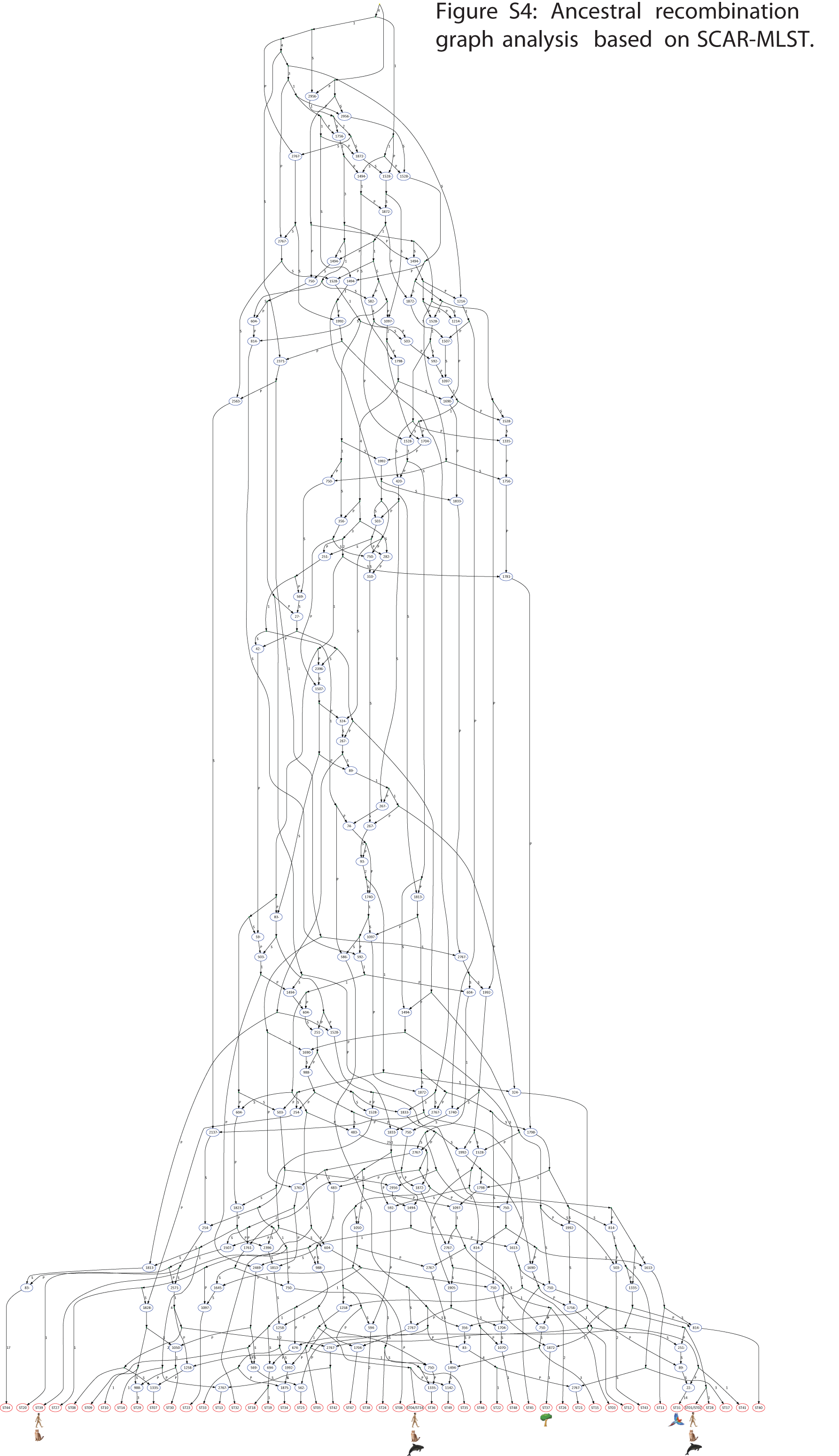

Supplement: Figure S4 — Ancestral recombination graph analysis based on SCAR-MLST. The ancestral recombination graph shows historical recombination events that gave rise to the current population structure represented by the clone corrected 49 sequence types (STs) observed within the concatenated nuclear SCAR-MLST dataset (Table S3). The ST01/ST02 lineage represents strains with the major Vancouver Island outbreak genotype AFLP6A/VGIIa (indicated with a human, cat and dolphin symbol to represent the clinical and veterinary strains), similarly indicated is ST04 representing the minor genotype AFLP6B/VGIIb. The recently emerged C. gattii AFLP6C/VGIIc Pacific Northwest outbreak (ST39) has been indicated with a human and cat symbol. The tree icon indicates the most ancestral lineage that was isolated from a tree in the pristine Amazon rainforest (ST26). The 167 blue circles indicate historical recombination breakpoint positions within the concatenated nuclear SCAR-MLST dataset. (TIF) [file pone.0071148.s004.tif]

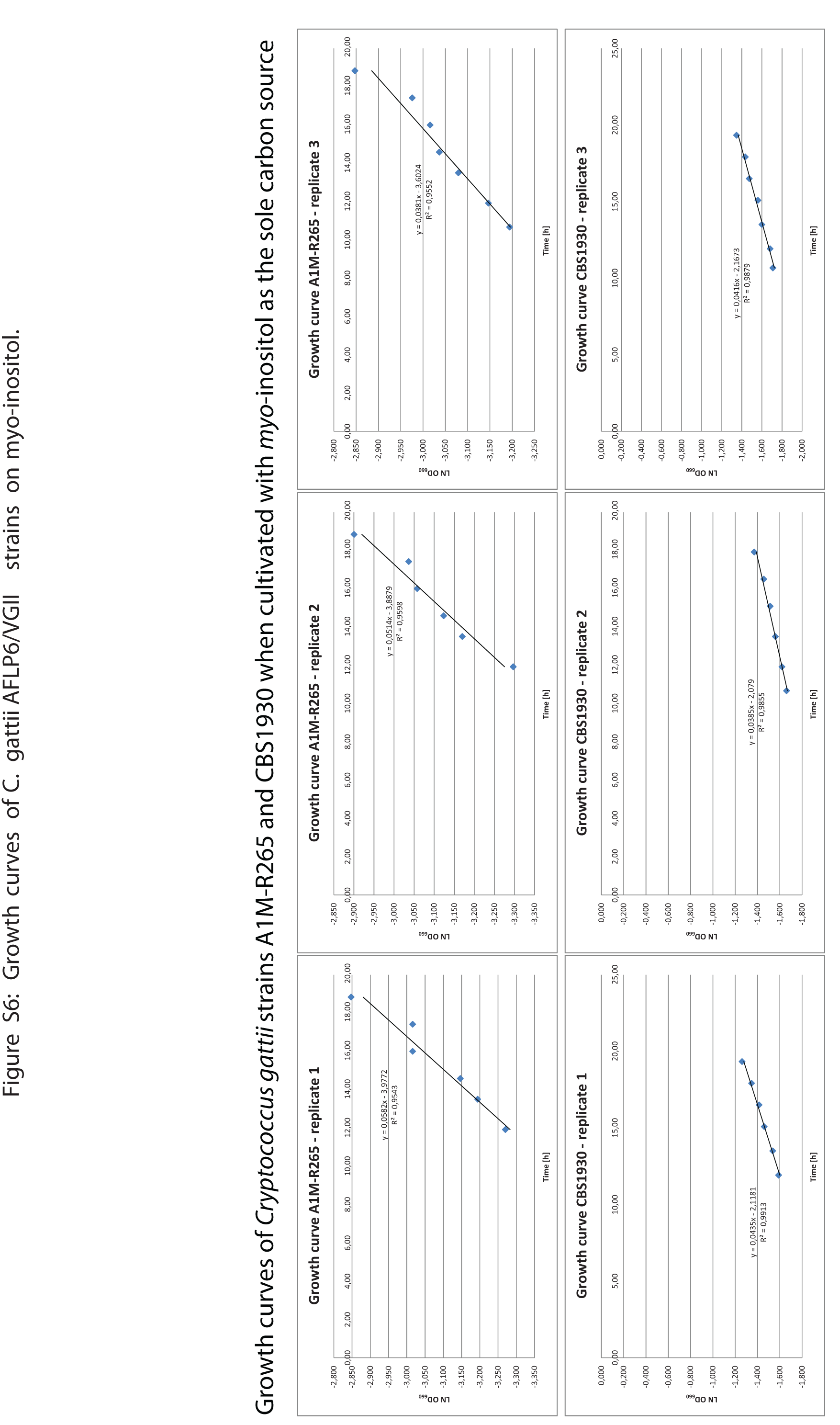

Supplement: Figure S6 — Growth curves of C. gattii AFLP6/VGII strains on myo -inositol. Growth curves of the three replicated growth rate experiments for A1M-R265 (major genotype AFLP6A/VGIIa Vancouver Island outbreak lineage) and CBS1930 (Aruba). The formula for each of the trendlines is provided as this is part of the formula to calculate the generation time. For the coalescence gene genealogy generation time scale calculation, the average value is used from all six growth rate experiments. (TIF) [file pone.0071148.s006.tif]
