# Supplementary material for: Ancient Dispersal of the Human Fungal Pathogen Cryptococcus gattii from the Amazon Rainforest
Source: PLoS One. 2013 Aug 7;8(8):e71148. doi: 10.1371/journal.pone.0071148 (PMC3737135; doi:10.1371/journal.pone.0071148)
Supplement: Table S1 — Map of informative sites used for the coalescence gene genealogy analysis. The clone corrected SCAR-MLST data has been collapsed into haplotypes after removal of homoplasious sites. Informative sites within the complete dataset, and among the 32 haplotypes identified, are provided per nuclear SCAR-MLST locus that has been provided as the Fragment-number. IGS1 refers to the Intergenic Spacer 1 region (see Table S5). Colours used for each of the loci correspond with those used to mark the mutation events along the coalescence gene genealogy in Fig. 1. (PDF) [file pone.0071148.s007.pdf]

Table S1: Map of informative sites used for the coalescence gene genealogy analysis.

Fragment 15 | Fragment 32 | F34 | Fragment 54 | IGS1

```
Position                                                            1111111111111111111111111122222222222222222222222222222222222333333
                                  1112222222233333344556667900000001111112224444788990000001122222222344444555556668899900001 2
                        1111112266347011234560134460645799101225899333346148345743571701345623113337791224581225534807027134981
                  2346891345890267309369192596536762424415562563306678957092213340547589519126376783926076513157997271524362545

Site Number                                                                                                             1111111111
                           1111111111222222222233333333334444444444555555555566666666667777777777888888888899999999990000000000
                  1234567890123456789012345678901234567890123456789012345678901234567890123456789012345678901234567890123456789

Consensus         GGTGTGGCTAATACATAAATTTCAACCAGCGTATGCCCGTGGGCAGTGTCGCATCCAACATTTCTTCGGGACAATAAGCGTCCCTGAGTTCCCTATGCAGGTTGTGTGC
Site Type         vtvvtvvvvttttvvtvtvvvtttttttvttvtvvtvttttttvtvvtvttttttttttvtttttttttttttvvvttttvvvtttvtvvtvtvttvtvvtvvtttttt
Character Type    ---------------ii-ii----ii--i-iiii-i-i-ii-i--i-iiiiii-----ii----iii-ii--iii-iiiiiiiiiiiiiii-------i-i-------i
H1          ( 42) ..............C.........G...TTAAG..........................................................................
H2          (  1) TAGCCTCAAGGCGA..........................................................................................C..........
H3          ( 41) .............................................................................................................
H4          (  1) ...............CC..A.......................A.........................A........................................
H5          (  1) ...............CC..A.......................A.........................A.............................T...........
H6          (  1) .................G.........................A............TG.TG...............................................
H7          (  3) ..................T......T...................................................................................
H8          (  1) ..................T......T..........G........................................................................
H9          (  7) ....................AC....TG..............A..........CT...TG.CC..............................G.T..C.AG...CA.
H10         (  1) ......................T......................................................................................
H11         (  2) .......................GG...T.AAG..............................................C...............A.................
H12         (  8) ........................G...T......T...CA......A..............T.............................................
H13         (  1) ........................G...T....A.................................G......................G...............
H14         (  1) ........................G...T....A...........................................................................
H15         (  1) ........................G...T......T...CA......A.............................................................
H16         (  1) .........................T...................................................................................
H17         (  1) ..................................T...A......T..G.ATG...........CCT..A..GTG.GATAAAATCACAAAT..................
H18         (  1) .....................................T.......................................................................C....
H19         ( 27) .....................................T.......................................................................
H20         (  5) ..........................................A.................................................T................
H21         (  4) ...........................................A..............................................................A...
H22         (  1) ...........................................A.................................................................
H23         (  5) ............................................AG....T.........A...............................................
H24         (  2) ..............................................T..G.ATG...........CCT..A..GTG.GATAAAATCACAAAT..................
H25         (  1) ................................................A.........................................................A..A.....
H26         (  4) ..................................................T..........................................................
H27         (  5) .........................................................G......CCTA.A..GTG.GATAAAATCACAAAT..................
H28         (  1) ................................................................CCT..A..GTG.GATAAAATCACAAAT..................
H29         (  2) .......................................................................T.............................A.......|
H30         (  2) ...............................................................................................C..C..........
H31         (  3) ....................................................................................................A.......|
H32         (  1) ....................................................................................................A........
```
