# Supplementary material for: Ancient Dispersal of the Human Fungal Pathogen Cryptococcus gattii from the Amazon Rainforest
Source: PLoS One. 2013 Aug 7;8(8):e71148. doi: 10.1371/journal.pone.0071148 (PMC3737135; doi:10.1371/journal.pone.0071148)
Supplement: Table S2 — Population sizes and historical migration rate estimates among Cryptococcus gattii populations. The population sizes and migration rates were estimated using Migrate v2.3 (http://popgen.scs.fsu.edu) based on the five nuclear SCAR-MLST loci. The centre column shows the mean Nmµ value, while the left flanking values represents the lower (left value) and upper (right value) 95% confidence interval values. (PDF) [file pone.0071148.s008.pdf]

Table S2: Population sizes and historical migration rate estimates among *Cryptococcus gattii* populations. The population sizes and migration rates were estimated using Migrate v2.3 (http://popgen.scs.fsu.edu) based on the five nuclear SCAR-MLST loci. The centre column shows the mean Nmμ value, while the left flanking values represents the lower (left value) and upper (right value) 95% confidence interval values.

**Population**	**Population size (θ)** [2Nmμ]			**2Nm [+=receiving population]**														
				Lower SA,+	Mean SA,+	Upper SA,+	Lower AFR,+	Mean AFR,+	Upper AFR,+	Lower AUS,+	Mean AUS,+	Upper AUS,+	Lower EUR,+	Mean EUR,+	Upper EUR,+	Lower NA,+	Mean NA,+	Upper NA,+
**South America (*n* = 76)**	0,0121	0,0224	0,0485				0,0000	0,0000	1,1954	2,8948	6,3345	11,7904	0,1631	2,8550	12,5672	0,0000	0,0000	0,3214
**Africa (*n* = 10)**	0,0030	0,0094	0,0568	0,0000	0,0000	2,9298				0,0000	0,0000	1,5210	0,1631	2,8550	12,5672	0,2979	0,8292	1,7763
**Australasia (*n* = 38)**	0,0024	0,0062	0,0249	6,5686	12,2932	20,6754	0,0000	0,0000	1,1954				0,1631	2,8550	12,5672	0,0000	0,0000	0,3183
**Europe (*n* = 6)**	0,0022	0,0098	0,1712	0,7640	3,0733	7,9845	0,0000	0,0000	1,1954	0,0000	0,0000	1,5210				0,0000	0,0000	0,3183
**North America (*n* = 48)**	0,0061	0,0089	0,0137	6,1579	11,9553	20,5466	1,9000	4,3769	8,3399	1,8882	4,7509	9,6257	3,5451	11,4201	26,5310			
