# Supplementary material for: Ancient Dispersal of the Human Fungal Pathogen Cryptococcus gattii from the Amazon Rainforest
Source: PLoS One. 2013 Aug 7;8(8):e71148. doi: 10.1371/journal.pone.0071148 (PMC3737135; doi:10.1371/journal.pone.0071148)
Supplement: Table S4 — Overview of molecular variation, including number of haplotypes, nucleotide diversity, estimates of theta based on the number of segregating sites and recombination parameters. The genetic diversity is provided per population and for all strains, as well as for each locus independently and the mean value for the given values. Given values are for the number of sites within an alignment with or without alignment gaps, the nucleotide diversity per site (π) corresponding to the average number of nucleotide differences per site between two sequences, the number of segregating or polymorphic sites (S), the Watterson’s estimated θ per locus and per nucleotide, the ‘Hud4Nc per site’ value representing the recombination rate per generation between the most distant nucleotides. The last two values listed the number of recombination events based on the ‘four gametic test’ and the minimum number of recombination events in the history of the sample (Note that provides an underestimation of the number of recombination events). (PDF) [file pone.0071148.s010.pdf]

Table S4: Overview of molecular variation, including number of haplotypes, nucleotide diversity, estimates of theta based on the number of segregating sites and recombination parameters. The genetic diversity is provided per population and for all strains, as well as for each locus independently and the mean value for the given values. Given values are for the number of sites within an alignment with or without alignment gaps, the nucleotide diversity per site (π) corresponding to the average number of nucleotide differences per site between two sequences, the number of segregating or polymorphic sites (S), the Watterson's estimated θ per locus and per nucleotide, the 'Hud4Nc per site' value representing the recombination rate per generation between the most distant nucleotides. The last two values listed the number of recombination events based on the 'four gametic test' and the minimum number of recombination events in the history of the sample (Note that provides an underestimation of the number of recombination events).

Groups of populations	Loci	Fragment size (including/ excluding gaps) (nt)		Number of haplotypes	Nucleotide diversity per site (π)	Segregating sites (S)	Theta per gene, from S	Theta per site, from S (μ)	Hud4Nc per site	Number of pairs of sites with four gametic types	Minimum number of recombination events
**Africa ($n_{STRAINS}$ = 10)**											
	F15	714	707	3	0.01226	13	8.667	0.0123	0.0167	-	-
	F32	604	601	3	0.00666	6	4.000	0.0067	n/a	-	-
	F34	594	593	3	0.01012	9	6.000	0.0101	0.3125	-	-
	F54	717	714	2	0.03641	26	26.000	0.0364	0.2404	-	-
	IGS	728	722	3	0.00554	6	4.000	0.0055	0.3700	-	-
	Mean	671	667	3	0.01420	12	9.733	0.0142	0.2349	-	-
**Australasia ($n_{STRAINS}$ = 38)**											
	F15	714	707	6	0.01905	33	13.467	0.0190	0.0092	4	1
	F32	604	601	4	0.01276	15	7.667	0.0128	n/a	-	-
	F34	594	593	6	0.01518	21	9.000	0.0152	0.0260	8	3
	F54	717	715	3	0.00466	5	3.333	0.0047	0.0731	-	-
	IGS	728	700	5	0.00571	10	4.000	0.0057	0.0052	-	-
	Mean	671	663	5	0.01147	17	7.493	0.0115	0.0284	2	1
**Europe ($n_{STRAINS}$ = 6)**											
	F15	714	707	4	0.01532	21	10.833	0.0153	0.0302	-	-
	F32	604	601	6	0.00588	10	3.533	0.0059	0.2781	-	-
	F34	594	593	5	0.01518	9	4.400	0.0074	n/a	5	2
	F54	717	715	4	0.0049	7	3.500	0.0049	0.1569	-	-
	IGS	728	722	5	0.0036	6	2.600	0.0036	n/a	-	-
	Mean	671	668	5	0.00898	11	4.973	0.0074	0.1551	1	-
**North America ($n_{STRAINS}$ = 48)**											
	F15	714	705	5	0.01617	20	11.400	0.0162	0.0023	-	-
	F32	604	601	3	0.00555	5	3.333	0.0055	n/a	-	-
	F34	594	593	3	0.01012	9	6.000	0.0101	0.1226	-	-
	F54	717	715	2	0.00699	5	5.000	0.0070	n/a	-	-
	IGS	728	722	3	0.00185	2	1.333	0.0018	n/a	-	-
	Mean	671	667	3	0.00814	8	5.413	0.0081	0.0625	-	-
**South America ($n_{STRAINS}$ = 76)**											
	F15	714	706	19	0.02031	60	14.339	0.0203	0.0431	189	14
	F32	604	597	19	0.00544	27	4.994	0.0084	0.0308	1	1
	F34	594	593	18	0.01063	24	6.301	0.0106	0.2077	47	7
	F54	717	714	9	0.01113	31	7.944	0.0111	0.0000	-	-
	IGS	728	711	17	0.00325	14	2.309	0.0032	1.1310	-	-
	Mean	671	664	16	0.01015	31	7.177	0.0107	0.2825	47	4
**All populations combined ($n_{STRAINS}$ = 178)**											
	F15	714	705	37	0.01933	66	13.631	0.0193	0.0294	215	13
	F32	604	597	21	0.00908	31	5.419	0.0091	0.0433	7	2
	F34	594	593	25	0.01188	28	7.047	0.0119	0.1380	103	8
	F54	717	714	11	0.01431	33	10.218	0.0143	0.0000	-	-
	IGS	728	689	22	0.00396	21	2.727	0.0040	0.0169	-	-
	Mean	671	660	23	0.01171	36	7.808	0.0117	0.0455	65	5
