# Supplementary material for: Ancient Dispersal of the Human Fungal Pathogen Cryptococcus gattii from the Amazon Rainforest
Source: PLoS One. 2013 Aug 7;8(8):e71148. doi: 10.1371/journal.pone.0071148 (PMC3737135; doi:10.1371/journal.pone.0071148)
Supplement: Table S5 — Primers used for SCAR-MLST and microsatellite typing. (PDF) [file pone.0071148.s011.pdf]

Table S5: Primers used for SCAR-MLST and microsatellite typing

Loci included in the SCAR-MLST	Primer name	Primer sequence	Reference	Nucleotide Substitution model[A][B]
"Fragment 15" - Non-coding region at supercontig 5	F15 - Forward	TAAgCTggAACAgCCAAgCA	This study	K80 I+G[A]
	F15 - Reversed	ggTAAgTgggTCgTAAgTAggA	This study	
	F15 - Long Forward	CTCAATTgTACCCTCACgAA	This study	
	F15 - Long Reversed	TgTgggAAAgTgAATCggAA	This study	
"Fragment 32" - Non-coding region at supercontig 4	F32 - Forward	AgTgATAgCTCCCATTCAgCA	This study	HKY G[B]
	F32 - Reversed	gACTCTggTTAgAgggAACTgA	This study	
	F32 - Long Forward	TATTgAgATCTgAgCTCCTCCCAA	This study	
	F32 - Long Reversed	AgCATTTgCCTTgTAgTggCAA	This study	
"Fragment 34" - Putative glutamate carboxypeptidase gene at supercontig 1	F34 - Forward	CgATCCCAAgTTCCTCCA	This study	K80 I+G[A]
	F34 - Reversed	AAgCTCAAAgggAgggTA	This study	
"Fragment 54" - Partly sodium:inorganic phosphate symporter gene at supercontig 12	F54 - Forward	CCTTggCTgggTCTTgACA	This study	K80[A]
	F54 - Reversed	TgTgCTgTCTgATTgggAgA	This study	
	F54 - Long Forward	TAgCCACACAggTTgCTgA	This study	
	F54 - Long Reversed	CCTCTTCACCTTCAAACTTCCA	This study	
Inter-Genic Spacer (IGS1)	IGS1 - Forward	CAgACgACTTgAATgggAACg	Bovers et al., 2008[D]	HKY G[B]
	IGS1 - Reversed	ATgCATAgAAAgCTgTTgg	Bovers et al., 2008[D]	
	IGS1 - Forward (sequencing only)	CTgCTggTgCTTgAgTTgCA	This study	
	IGS1 - Reversed (sequencing only)	ATgCAACTCAAgCACCAgCA	This study	

Loci included in the extended 'Fraser & Byrnes' MLST	Primer name	Primer sequence	Reference	Nucleotide Substitution model[A][B]
CAP59 - Capsular Associated Protein	JOHE14386	CCggAACTgACCACTTCATC	Litvinseva et al., 2005[F]	HKY G[B]
	JOHE14387	gCCCACTCAAgACACAACCT	Litvinseva et al., 2005[F]	
GPD1 - Glyceraldehyde-3-Phosphate Dehydrogenase	JOHE14408	CCACCgAACCCTTCTAggATA	Fraser et al., 2005[E]	HKY[B]
	JOHE14409	CTTCTTggCACCTCCCTTgAg	Fraser et al., 2005[E]	
IGS1 - Inter-Genic Spacer region 1	JOHE14968	ATCCTTTgCAgACgACTTgA	Litvinseva et al., 2005[F]	HKY G[B]
	JOHE14969	gTgATCAgTgCATTgCATgA	Litvinseva et al., 2005[F]	
LAC1 - Laccase	JOHE14970	AACATgTTCCCTgggCCTgTg	Fraser et al., 2005[E]	K80 G[A]
	JOHE14971	ATgAgAATTgAATCgCCTTgT	Fraser et al., 2005[E]	
MPD1 - Mannitol-1-Phosphate Dehydrogenase	JOHE14972	TgCCCTggATCCTAATgCTCT	Fraser et al., 2005[E]	HKY G[B]
	JOHE14973	ACCCAgACTgCCgCTgTCgTC	Fraser et al., 2005[E]	
PLB1 - Phospholipase B	JOHE14974	CTCTCATTgTTCgCCgCTACT	Fraser et al., 2005[E]	K80[A]
	JOHE14975	ggAAgCCgAggTCTgATTTgg	Fraser et al., 2005[E]	
TEF1 - Translation Elongation Factor sub-unit 1α	JOHE14976	gCACgCTCTTCTCgCCTTCAC	Fraser et al., 2005[E]	GTR I[C]
	JOHE14977	gTAgTCggCgTAggTCTCAAC	Fraser et al., 2005[E]	

(A), (B), (C), Optimal nucleotide substitution model estimated using MrModeltest 3.7 (see Material and Methods section). "I" indicates "with Invariant sites" and "G" indicates "Gamma distributed"

(A) Kimura M. A simple method for estimating evolutionary rates of base substitutions through comparative studies of nucleotide sequences. J Mol Evol. 1980;16:111-120.

(B) Hasegawa M, Kishino H, Yano T. Dating of human-ape splitting by a molecular clock of mitochondrial DNA. J Mol Evol. 1985;22:160-174.

(C) Tavaré S. Some probabilistic and statistical problems in the analysis of DNA sequences. Lect Math Life Sci. 1985;17:57-86.

(D) Bovers M, Hagen F, Kuramae EE, Boekhout T. Six monophyletic lineages identified within Cryptococcus neoformans *and* Cryptococcus gattii *by multi-locus sequence typing. Fungal Genet Biol. 2008;45:400-421.*

(E) Fraser JA, Giles SS, Wenink EC, Geunes-Boyer SG, Wright JR, Diezmann S, Allen A, Stajich JE, Dietrich FS, Perfect JR, Heitman J. Same-sex mating and the origin of the Vancouver Island Cryptococcus gattii *outbreak. Nature. 2005;437:1360-1364.*

(F) Litvintseva AP, Thakur R, Vilgalys R, Mitchell TG. Multilocus sequence typing reveals three genetic subpopulations of Cryptococcus neoformans *var.* grubii *(serotype A), including a unique population in Botswana. Genetics. 2006;172:2223-2238.*

Microsatellite primers for ***Cryptococcus gattii*** **AFLP6**		Primer name (fluorescent label)	Primer sequence	Reference
CNB2 Panel 1	CNB2-16 (TC repeat)	CNB2_16A (FAM)	TAgCTCATCCCCgATTTTTg	This study
		CNB2_16B	gCCAgAgggTTTgCTAAgAg	This study
	CNB2-17 (AT repeat)	CNB2_17A (HEX)	AACgAAgACgCCAAAACAAT	This study
		CNB2_17B	gCACTTgAgACCgTCCCCTA	This study
	CNB2-18 (AT repeat)	CNB2_18A (TAMRA)	CACTgCggATATTTggATCA	This study
		CNB2_18B	gAAggCACATCTCCgATCTTg	This study
CNB2 Panel 2	CNB2-22 (TA repeat)	CNB2-22A (FAM)	CCATTggTgTTTTCTCATCTCC	This study
		CNB2-22B	gCAAAAgCTgTTggCTgTTAgC	This study
	CNB2-23 (AT repeat)	CNB2-23A (HEX)	gCAgACgAAgAATggTggAT	This study
		CNB2-23B	ggAgAAgggTggTggATTATg	This study
	CNB2-24 (AT repeat)	CNB2-24A (TAMRA)	TTTACTgCTCTgACgATggAg	This study
		CNB2-24B	gAgAgAAgAAggCCgAAACA	This study
CNB3 Panel 3	CNB3-12 (CTT repeat)	CNB3_12A (TAMRA)	TATCAgACCCgggAAACAAg	This study
		CNB3_12B	gAgATCgACgACgCTTgTAA	This study
CNB6 Panel 4	CNB6-4 (CCTCTg repeat)	CNB6_4A (FAM)	AggCTCgTCAgAgTCggTTA	This study
		CNB6_4B	gggCAgAAgAAgACgAAgAA	This study
	CNB6-5 (gCAggA repeat)	CNB6_5A (HEX)	ACTTTgTggACgAggAgCAT	This study
		CNB6_5B	gCCACTTCTgAACCCAgTgT	This study
	CNB6-6 (TCATAC repeat)	CNB6_6A (TAMRA)	CAgAgggATgAgTgggAgTT	This study
		CNB6_6B	gTgCACgTgAgAgAAAgATCg	This study
