# Supplementary material for: Ancient Dispersal of the Human Fungal Pathogen Cryptococcus gattii from the Amazon Rainforest
Source: PLoS One. 2013 Aug 7;8(8):e71148. doi: 10.1371/journal.pone.0071148 (PMC3737135; doi:10.1371/journal.pone.0071148)
Supplement: Materials and Methods S1 — Supporting Information. (DOCX) [file pone.0071148.s012.docx]

**Supplementary Information**

**SI Material & Methods­­**

*Analysis of generation time of* C. gattii *AFLP6/VGII strains*

Strains were cultivated for 48h on malt-extract agar at 25°C. One colony of each strain was suspended in 100ml Yeast Nitrogen Base (Difco, BD, Breda, The Netherlands) supplemented with *myo*-inositol, according to the manufacturer’s instructions, and cultured for 48h at 25°C under agitation (150rpm). The carbon source *myo*-inositol, typically assimilated by tremellaceous yeasts, was chosen for the generation experiment since this is an important factor for the sexual development and virulence potential of *Cryptococcus*, and it is abundantly present in the human central nervous system [S1,S2]. Erlenmeyer flasks (300ml) containing 150ml Yeast Nitrogen Base (Sigma Aldrich, St. Louis, MO) liquid medium, supplemented with 2% *myo*-inositol, were inoculated with 1.5ml liquid pre-culture and incubated for 48h at 25°C under agitation (150rpm). Directly after inoculation, 400µl was used to measure OD_660_ spectrophotometrically followed by hourly measurements starting 7h after inoculation. Cell concentrations at different time points were counted using a Bürker-Türker counting chamber to determine the increase in cell density.

To calculate the generation time, the natural log of the OD_660_ was plotted against the time (hours) in Excel 2007 (Microsoft, Redmond, WA). Data-points before and after the log-growth phase were removed from the curve and the equation (y = µ · x + b) and the R^2^ were calculated using a trendline through the remaining data-points. The generation time (*g*) could then be derived when the natural log of µ was calculated, which revealed the number of hours necessary for one *C. gattii* strain to replicate once under the given circumstances. The number of *C. gattii* generations per year is the number of hours per year (8760h) divided by *g*. The generation-time for *C. gattii* per year (*n*) was determined by cultivating the strains A1M-R265 and CBS1930 in triplicate which resulted in 619 ± 128 and 514 ± 32 generations per year, respectively, with an average generation time of 567 ± 93 generations per year (Fig. S6).

*Ploidy analysis*

The ploidy of selected *C. gattii* strains (Table S3) was analyzed according to the method described by Bovers et al. [S3]. The two haploid reference strains A1M-R265 and A1M-R272 and the homozygote diploid *C. gattii* AFLP6A/VGIIa strain RB59JF were included [S4]. The nuclear content of 30 *C. gattii* AFLP6/VGII strains was investigated using flow cytometry analysis (Table S3). All strains were found to be haploid, compared to the two reference strains and the diploid strain RB59JF. An independent sub-culture of the strain RB59 (designated RB59FH in the current study) was found to be haploid whereas another sub-culture was confirmed to be diploid (designated in the current study as RB59JF) as originally reported and received from another research group [S4]. Another sub-culture of this strain (RB59WM), which was sent from the original distributor at the same time to a third research group, was subsequently found to be haploid. The sub-culture of RB59JF may have become diploid via different processes, such as autodiploidization, same-sex mating, or cell-cell fusion [S5].

*DNA extraction*

Genomic DNA was extracted as described previously [S12]. Briefly, cultures were allowed to grow for 48h at 25°C on YPGA-medium supplemented with 0.5M sodium chloride to prevent capsule formation. Approximately 150µl of cells were harvested and dissolved in 1.6ml urea buffer and incubated at room temperature for 3h. Cells were lysed by bead beating at 2500rpm for 3min in phenol-chloroform-isoamylalcohol (25:24:1; pH 8.0). Genomic DNA was precipitated with ice-cold EtOH 96% and 100µl ammonium acetate 3.0M and afterwards dissolved in 100µl TE-buffer (pH 8.0). Genomic DNA was purified using a GFX™ PCR DNA and Gel Band Purification Kit (Amersham Biosciences, Piscataway, NJ).

*Mating-type determination*

Partial amplification of the *STE12* gene, as described by Bovers et al. [S3], was performed to determine mating-type **a** and α specific regions of all strains using *C. gattii* strains CBS1930 (**a**B) and CBS6956 (αB) as reference strains.

*AFLP fingerprinting*

Amplified fragment length polymorphism (AFLP) analysis was carried out in duplicate on an ABI Prism 310 Genetic Analyzer platform (Applied Biosystems, Foster City, CA) as described previously [S6,S7] using the following four combinations of selective primers: *Eco*R1-AC/*Mse*I-G, *Eco*R1-AC/*Mse*I-C, *Eco*R1-AG/*Mse*I-G and *Eco*R1-AT/*Mse*I-G. A set of five *C. neoformans* (WM148, WM626Brown, WM626White, WM628 and WM629) and four *C. gattii* strains (WM161, WM178, WM179 and WM779) were used as a reference (Table S3). The presence and absence of specific loci in the four duplicated AFLP patterns were scored manually, according to Campbell et al. [S8]. The distance between strains was calculated with PAUP4.0.10b (Sinauer Associates Inc., Sunderland, MA) using the Neighbour Joining (BioNJ method) algorithm. For this analysis, ties were broken randomly and, when characters observed to be different, the ‘mean character difference’ was used. The restriction-site distance was calculated using uphold and with ‘among site variation’.

*Multi-locus sequence typing approaches*

The sequence characterized AFLP regions multi-locus sequence typing (SCAR-MLST) scheme was developed based on sequenced polymorphic markers obtained by differential AFLP fingerprinting analysis [S9]. Twelve strains, indicated in Table S3, were selected from the phylogenetic analysis based on the AFLP matrix obtained as described above. Fragments were excised from the polyacrylamide gel and the selective primers from the initial AFLP-selective PCR were used to generate amplicons from these excised fragments. The amplicons were purified using the GFX™ PCR DNA and Gel Band Purification Kit (Amersham Biosciences, Piscataway, NJ), followed by agarose gel electrophoresis to check quantity and quality.

Sequencing of the excised AFLP fragments was carried out using the BigDye v3.1 Chemistry kit (Applied Biosystems, Foster City, CA) using unlabeled primers identical to those used for the selective AFLP PCR. Sequencing reactions were carried out as described by Bovers et al. [S10]. Raw data was automatically aligned using SeqMan version 5.03 (DNAStar, Madison, WI) and ambiguously placed nucleotides were manually corrected.

AFLP fragment consensus sequences were compared to the available genomes of *C. gattii* strain A1M-R265 (serotype B; The Broad Institute; <http://www.broad.mit.edu/>; [S11]) and the annotated genomes of *C. neoformans* var. *neoformans* strains JEC21 and B3501 (serotype D; TIGR The Institute for Genomic Research, <http://www.tigr.org/> and The Stanford Genome Technology Center; <http://www-sequence.stanford.edu/> respectively) [S12] using the local BLAST program (available at <ftp://ftp.ncbi.nlm.nih.gov/blast/>).

Fragments that corresponded to known functional categories of genes, or that had high homology with predicted proteins, were used as starting-point for primer design using GeneFisher [S13]. Primers were designed, based on the genome of *C. gattii* strain A1M-R265, at the end of the approximately 350bp long flanking regions of the sequenced original fragment, with a maximum length of 25 nucleotides and a melting temperature between 50 to 65°C (Table S5). A subset of twelve *C. gattii* strains, indicated in Table S3, was used to amplify the obtained polymorphic markers and thereby identifying the fragments with the highest proportion of polymorphic sites. Amplicons were sequenced and analyzed as described above, resulted in the SCAR-MLST dataset that comprised five nuclear loci. Sequence type numbers were assigned using the non-redundant database software (<http://www.mlst.net/>). Sequences were deposited in Genbank with the accession numbers HM997344-HM998285 and HQ706505-HQ706637.

Additionally, a set of 36 strains (Table S3) were used to supplement the MLST dataset of Byrnes et al. [S14] and Fraser et al. [S4]. The *MPD1*-locus was excluded because it contained no polymorphic sites among AFLP6/VGII strains [S4]. Additional strains came from Europe (*n* = 4) and South America (*n* = 32). Amplification of these loci was performed as described by Fraser et al. [S4] and sequence analysis was carried out as described above. Sequence type numbers were assigned according to those published by Fraser et al. [S4] and Byrnes et al. [S14] and new ST numbers were assigned to novel sequence types as detected by the non-redundant database software (<http://www.mlst.net/>). In the current study, we refer to this dataset as the extended ‘Fraser and Byrnes’ MLST dataset to distinguish it from the above developed SCAR-MLST dataset.

*Microsatellite analysis*

Microsatellite typing involved a panel of ten microsatellite markers consisting of six dinucleotide repeat markers, one trinucleotide repeat markers and three hexanucleotide repeat markers. Primer sequences are indicated in Table S5. All markers were amplified in four multicolor multiplex amplification reactions (containing three markers each, except for the trinucleotide-repeat marker). Amplification was performed by applying an initial denaturation step for 10 min at 95°C followed by 30 cycles of 30s at 95°C, 30s at 60°C and 60s at 72°C. An additional incubation for 10 min at 72°C was applied before the reactions were cooled down. Reaction mixtures contained 1U FastStart Taq DNA polymerase (Roche Diagnostics, Almere, The Netherlands), 1 ng DNA, 10 pmol of each primer, 0.2 mM dNTP’s and 3 mM MgCl_2_ in a total volume of 50 µl of 1× PCR reaction buffer. Following amplification, PCR products were analyzed on a MegaBACE 500 platform (GE Healthcare, Diegem, Belgium) equipped with a 48 capillary array as described by Illnait-Zaragozi et al. [S15]. Repeat numbers were determined by comparing the relative size of the obtained fragments to those obtained using the reference strain A1M-R265 whose genome has been elucidated [S11]. Microsatellite repeat numbers are listed in Table S3. Occasionally, non-integer alleles were obtained as the result of the presence of indels in the flanking region of the repeat region. The identity of the indels and verification of repeat numbers in selected alleles was performed by subcloning amplicons in a pJET1.2 cloning vector (Fermentas, St. Leon-Rot, Germany) followed by direct sequencing, as described above, of the microsatellite insertion from the purified plasmid DNA. Repeat numbers in non-integer alleles were rounded off to the appropriate integer according to the identity of the indel. Using the repeat numbers for all loci, a minimum spanning tree was generated with BioNumerics version 4.6 (Applied Maths, Sint-Martens-Latem, Belgium) using a categorical analysis and UPGMA clustering. Microsatellite complexes (MCs) were determined to have a minimum size of two types and with a maximum neighbor distance of two loci among the ten studied.

*Phylogenetic sequence analysis*

Multiple sequence alignments were generated in MEGA version 5 [S16] with the ClustalW algorithm. Loci were concatenated using FSorter, a script written and executable in Python (<http://www.python.org/>).

For phylogenetic analysis, the best fitting nucleotide substitution model was determined using MrModeltest v3.7 (<http://www.abc.se/~nylander/>), observed nucleotide substitution models are listed in Table S5. MEGA v5 [S16] was used to perform a bootstrapped Maximum Likelihood phylogenetic analyses with 1000 iterations using the nucleotide substitution of Hasegawa-Kishino-Yano model with invariant sites and gamma distributed, which was found to be the best fitted model for the concatenated nuclear SCAR-MLST dataset.

*Genetic diversity*

Simpsons diversity index (*D*) was used to determine the discriminatory power of the AFLP, microsatellite and MLST data, as well as to assess the genetic diversity among the defined populations [S17]. For the calculation of the Stoddart and Taylor genetic diversity, isolates that shared an identical dAFLP, microsatellite typing or SCAR-MLST profile were treated as clones. Genotypic diversity was quantified as the number of different genotypes per dataset, and, secondly, using Stoddart and Taylor’s genotypic diversity, where pi is the frequency of the *i*th genotype [S18]. Pairwise bootstrap tests were performed to test whether pairs of populations differed in their genotypic diversity index using 1,000 permutations with subsampling to match the smallest population size using GenoDive v2.0b7 [S19]. The gene diversity was quantified based on allelic richness, which was calculated according to El Mousadik & Petit [S20] as the mean number of alleles per locus. A bootstrap approach based on 1,000 permutations was used to determine if groups of populations differed for allelic richness. Allelic richness and bootstrap tests were performed using the program FSTAT v2.9.3 [S21].

*Recombination analysis using DNASP*

To calculate the minimum number of recombination events (*R*_M_) the software package DnaSP v5.1 was used [S22-S24]. Recombination events were calculated as the minimum number of recombination events between two adjacent sites derived from the ‘four gametes test’, however, this is an underestimated number of recombination events [S22-S23]. DnaSP v5.1 was also used to calculate the Hudson-parameter ‘Hud4Nc per site’ according to Hudson [S22]. Next to these recombination tests, the nucleotide diversity per site (π) corresponding to the average number of nucleotide differences per site between two sequences, the number of segregating or polymorphic sites (S), the Watterson’s estimated θ per locus and per nucleotide (θ = 2Nm, with N being the effective population size and m the mutation rate per nucleotide per generation) were calculated using DnaSP v5.1. Results are listed in Table S4.

*Recombination analysis using the Pairwise Homoplasy Index*

The Pairwise Homoplasy Index, or Phi (Φ_W_) test, was used to distinguish recurrent mutations from recombination and was calculated for the complete concatenated datasets of the nuclear SCAR-MLST loci and the extended Fraser MLST (SplitsTree v4.7.18; [S25]).

*Recombination analysis using the CASS algorithm*

A reticulation network of the SCAR-MLST was calculated using the recently developed algorithm CASS [S26]. The five nuclear loci from the SCAR-MLST were separately analyzed and for each locus an unrooted phylogenetic tree was constructed using the implementation of the Neighbour Joining algorithm available in the SplitsTree v4 package [S27]. In each case the tree was rooted with strain LMM645 as this was the most basal lineage based on the coalescence gene genealogy. We calculated bootstrap values for each gene tree using 2000 bootstrap replicates. Subsequently, we selected those clades that had bootstrap support of at least 90%. Nine clades fulfilled this property. A phylogenetic network representing all these clades was constructed using the CASS algorithm, which is available as part of the software package Dendroscope v2 [S28].

*Ancestral recombination graph analysis*

To reconstruct the ancestral recombination graph of *C. gattii*, the sequence data from the complete set of five MLST markers was concatenated. Beagle was used to compute the minimum number of recombination events [S29] and to simultaneously reconstruct a minimal ancestral recombination graph using the Kwarg branch and bound algorithm implemented in Beagle [S29]. Kwarg implements a heuristic search, reducing computation time without significant loss of power [S29]. The reconstructed ARG is displayed in a hierarchically-directed graph, and can be rooted with the most recent common ancestor (MRCA) of the entire sample, inferred from coalescent simulations. The rooted ARG provides inferences on the relative order of recombination events and the contribution of mutation and recombination in the evolution of haplotypes.

*Inferring the coalescent history of mutations: neutrality tests, population subdivision analysis, migration and coalescence*

In order to reconstruct the history of the mutations in our data set, we used coalescent methods, which have strict assumptions, such as neutrality and absence of recombination signals (identified as incompatible homoplasious sites). Sixty-eight incompatible homoplasious sites were manually removed from the concatenated nuclear SCAR-MLST alignment, producing a dataset with no incompatibilities and 109 informative sites. The removal of sites caused a 35% reduction in haplotypes from 49 to 32. Neutrality tests were performed in DnaSP v5.1 [S24], Tajima’s *D* [S30], Fu and Li’s *D* and *F* [S31] were calculated. Deviations from neutrality could be a sign of selection or changes in the effective population sizes, such as population expansion or contraction. Analyses were performed for every locus as well as for each population independently. Neutral evolution for the nuclear loci was observed except for the IGS1 locus (*P* < 0.05).

*Coalescence gene genealogy analysis*

The coalescent gene genealogy was simulated under the scenario of population subdivision and distinct population sizes using SNAP Workbench v2[S32]. Historical migration matrices and θ values for the coalescent simulations were estimated previously with Migrate v3.1.6 (Table S2) [S33]. Estimates were obtained using two hundred independent replicate runs, each run with ten initial short chains, two final long chains and static heating schemes with four temperatures (1.0, 1.3, 2.6 and 3.9), swapping interval of one. Confidence intervals for θ and *M* were calculated using a percentile approach [S33]. Subsequently, we reconstructed coalescent SCAR-MLST haplotype genealogies containing the ages of mutations backwards in time and the generation time for the most recent common ancestor (GTMRCA) using Genetree v9.0 [S34,S35]. Ten initial simulations with ten million runs each were performed, starting from ten distinct random seed numbers, corresponding to 10 million runs each. The coalescent gene genealogies with the highest likelihood were chosen from the initial ten simulations and a second simulation with ten million iterations was performed. The topology of the coalescent gene genealogy with time scale in coalescent units was constructed and depicted using Genetree/Treepic [S34,S35]. Ages of mutations in real *C. gattii* AFLP6/VGII generation time scale were generated as follows: the coalescence generation time t_GT_ in generations was calculated as t_GT_ = ((t / µ) · n) · n, where t is the Genetree TMRCA estimate, µ is the overall mutation rate for *C. gattii* (2 · 10^-9^ substitutions per nucleotide per year; [S11,S36]) and n is the generation time of *C. gattii.* The generation time n for *C. gattii* was determined using *myo*-inositol as the sole carbon source for strain A1M-R265 (H01; mating-type α) and CBS1930 (H19; mating-type **a**) were 619 ± 128 and 514 ± 32 generations per year, respectively, and were not significantly different (*P* = 0.281). The estimated time scale for the genetic diversification should be regarded as minimum values, because they depend largely on the generation time, which was estimated under laboratory circumstances. Most likely, the generation time of *C. gattii* AFLP6/VGII under natural conditions might be longer as has been observed for *C. neoformans* when cultured in soil and tree debris [S37-S39].

*Whole Genome Sequencing of* C. gattii *strain R265*

The genome of the clinical and virulent strain A1M-R265, the reference strain for the major genotype of the Vancouver Island outbreak, was re-sequenced using Illumina technology. Genomic DNA was isolated with the EpiCentre MasterPureTM Yeast DNA Purification Kit according to a modified version of the instruction manual. Briefly, the strains were grown in liquid YPD media for 24 h at 25°C rotating at 20 rpm. Cells from 3 ml of culture were harvested by centrifugation at 17,000 × *g* for 5 minutes. Cells were lysed in 300 μl of Yeast Cell Lysis solution by mechanical disruption with 0.1 mm silica spheres (FastPrep Lysing Matrix; MP Biomedicals, Santa Ana, CA) twice for 30 seconds at 6,800 rpm in a Precellys24 (Aix-en-Provence, France) and incubation at 65°C for 15 minutes. Samples were cooled down on ice for 5 minutes and proteins removed by vortexing with 150 μl of MPC Protein Precipitation Reagent (Cambio, Cambridge, UK) and following centrifugation for 10 minutes at 17,000 × *g*. DNA was recovered with 500 μl isopropanol and centrifugation at 17,000 x g for 10 minutes. DNA was purified by RNase A treatment for 60 min at 37°C followed by phenol:chloroform (24:1) extraction and ethanol precipitation. DNA yield and quality was determined by spectrophotometry. Two microgram of genomic DNA was used for library preparation, DNA was fragmented to 150-500 bp using Covaris shearing and processed with the TruSeq DNA Sample Prep Kit (Illumina, San Diego, CA) according to the manufacturer’s instructions. Purification steps were performed with Agencourt AMPureXP magnetic particles (Beckham Coulter, Fullerton, CA) on a magnetic stand. The whole genome A1M-R265 was re-sequenced on an Illumina HiSeq2000 at the MRC Clinical Science Centre, Imperial College London (UK).

*Whole Genome Sequencing of* C. gattii *strain CBS7750*

Illumina paired-end libraries were generated using 5 micrograms genomic DNA, extracted as described previously [S6], from the environmental and non-virulent *C. gattii* strain CBS7750 following the Illumina Paired-End Sequencing Sample Preparation Guide (version September 2009). The resulting library was sequenced on one lane of an Illumina GAIIx flow cell following the Illumina Genome Analyzer User Guide (version Rev. A, August 2009). Base calling, raw and past-filter sequences were obtained using the Illumina GA Pipeline software CASAVA-1.6.0, generating 3.15 × 10^7^ past-filter 2 × 100 bp reads with an insert size of 211 ± 42 bp. This total yield of 6.36 × 10^9^ bp of past-filter data corresponds approximately to a 350 fold coverage of the *C. gattii* CBS7750 genome.

*MIRA mapping assembly of Illumina sequence reads*

The Mimicking Intelligent Read Assembly package MIRA v3.2.0rc2 [S40] was used to perform a mapping genome assembly. Because of memory limitations the data was reduced to twice 2 × 10^7^ randomly selected paired-end reads. Following command line parameters were used to perform the assembly: -job=mapping,genome,accurate,solexa (mapping assembly of genomic DNA fragments, most accurate setting, expect Illumina GAIIx data) COMMON_SETTINGS -SK:not=16 (use 16 cpu’s) SOLEXA_SETTINGS -LR:ft=fastq (input file is FASTQ) -CO:msr=no (do not merge reads that are 100% identical to the backbone) -GE:uti=no:tismin=100:tismax=500 (switch off template size checking when inserting reads into the backbone to allow to spot genome re-arrangements or indels, minimum and maximum distance paired-end reads may be away from each other are 100 and 500 respectively). The recently published genome of *C. gattii* strain A1M-R265 [S11] was used as a reference backbone for the mapping assembly. This mapping assembly resulted in 28 contigs with a total consensus of 17.5 Mbp and an N50 contig size of 1.1 Mbp. The average coverage for the assembly was 208 fold.

*SOAP denovo*

The Short Oligonucleotide Analysis Package SOAP denovo v1.04 was used to perform a *de novo* genome assembly. The following parameters were issued through a config file: avg_ins=211 (average insert size between paired-end reads is 211), asm_flags=3 (use reads both in contig and scaffold assembly), rank=1 (libraries with the same rank are used at the same time during scaffold assembly, superfluous because only one library was used in this assembly), pair_num_cutoff=3 (minimum 3 paired-end reads are required to connect two contigs or pre-scaffolds), map_len=32 (minimum alignment length between a read and a contig for a reliable read location is 32) while following parameters were issued through the command line: -K 51 (use K-mer size of 51) -R (use reads to solve tiny repeats) -d 1 (remove low-frequency K-mers with frequency no larger than 1) -D 2 (remove edges with coverage no larger than 2) -p 16 (use 16 cpu’s). This mapping assembly resulted in 637 contigs with a total consensus of 17.2 Mbp and an N50 contig size of 129 kbp.

*SNP and indel detection using Varscan*

The Mosaik aligner package v1.0.1388 was used to map the reads to the reference sequence, following the default instructions in the Mosaik manual. The Samtools package v0.1.7 was used to create a pileup file from the aligned reads and to create reference guided assembly consensus sequence files. The Varscan package v2.1 was used to detect single nucleotide polymorphism (SNP) and indel calls. Homozygotic SNP calls were generated using following parameters: --min-coverage 12 (minimum read depth of 12 at a position to make a call) --min-reads2 4 (minimum four supporting reads at a position to call variants) --min-var-freq 0.7 (minimum variant allele frequency threshold) --p-value 0.05 (default *P*-value threshold for calling variants). Heterozygotic SNP were called using the parameters --min-coverage 12 --min-reads2 4 --min-var-freq 0.3 --p-value 0.05. Homozygotic and heterozygotic indels were called with the same parameter set. Using above parameters, Varscan called 3777 homozygotic SNPs, 4969 heterozygotic SNPs, 8 homozygotic indels and 4307 heterozygotic indels.

*Genome-wide divergence time estimate*

To estimate the divergence time between two closely related outbreak strains the re-sequenced genomes of *C. gattii* strain A1M-R265 and CBS7750 were compared. The gene predictor software Augustus [S41] with a configuration file trained for the published *C.* *neoformans* genome [S12] was ran on the two newly assembled contigs of *C. gattii* strains A1M-R265 and CBS7750. Genome sequences were corrected by aligning the Illumina reads for each re-sequenced strain using Bowtie version 2.0.0.7 in ‘--very-fast-local’ mode [S42] and then using GATK version 2.2.2 [S43] to realign reads around putative indels and to call variants. Detected variants were further filtered by removing heterozygous variants, clusters of over 4 variants in 20bp and low quality variants ("QD < 2.0 || MQ < 40 || FS > 60.0 || HaplotypeScore > 13.0 || MQRankSum < -12.5 || ReadPosRankSum < -8.0), as recommended by GATK developers. Best reciprocal blast hits between *C. gattii* strains A1M-R265 and CBS7750 were computed at the protein level using blast [S43] with an E-value 1 × 10^-10^ and identity (80%) thresholds. Pairs of reciprocal hits were aligned using MUSCLE v3.7 [S44]. To remove unreably aligned columns, only blocks of seven residues containing no gaps were selected using trimAl v1.3 [S45]. Synonymous positions in the alignment were selected and back-translated to the corresponding codons, according to the CDSs, using trimAl v1.3 [S46]. Trimmed alignments shorter than 600 nucleotides were discarded. Substitutions over the 8,118,815 reliably aligned positions (representing 4,771 orthologous pairs) were counted and the number of effective mutations was estimated using the Jukes-Cantor correction [S47]. Outliers deviating over two standard deviations from the average substitutions per site were removed. Divergence time was derived then using the neutral mutation rate of 2 · 10^-9^ substitutions per nucleotide per year [S11]. Standard error of the estimate was inferred using the variance of substitution rates observed in the gene sample. Standard Error of the mean was 1,863 years and 95% confidence interval [33,903-41,207] years.

**SI References:**

S1. Wang Y, Liu T, Delmas G, Park S, Perlin D, *et al.* (2011) Characterization of two major inositol transporters and their role in cryptococcal virulence. Eukaryot Cell 10: 618-628.

S2. Xue C (2012) *Cryptococcus* and beyond - inositol utilization and its implications for the emergence of fungal virulence. PLoS Pathog 8: e1002869.

S3. Bovers M, Hagen F, Kuramae EE, Diaz MR, Spanjaard L, *et al.* (2006) Unique hybrids between the fungal pathogens *Cryptococcus* *neoformans* and *Cryptococcus* *gattii*. FEMS Yeast Res 6: 599-607.

S4. Fraser JA, Giles SS, Wenink EC, Geunes-Boyer SG, Wright JR, *et al*. (2005) Same-sex mating and the origin of the Vancouver Island *Cryptococcus* *gattii* outbreak. Nature 437: 1360-1364.

S5. Lin X, Litvintseva AP, Nielsen K, Patel S, Floyd A, *et al*. (2009) Diploids in the *Cryptococcus* *neoformans* serotype A population homozygous for the alpha mating type originate via unisexual mating. PLoS Pathog 5: e1000283.

S6. Hagen F, Illnait-Zaragozi MT, Bartlett KH, Swinne D, Geertsen E, *et al*. (2010) *In vitro* antifungal susceptibilities and amplified fragment length polymorphism genotyping of a worldwide collection of 350 clinical, veterinary, and environmental *Cryptococcus* *gattii* isolates. Antimicrob Agents Chemother 54: 5139-5145.

S7. Boekhout T, Theelen B, Diaz M, Fell JW, Hop WCJ, *et al*. (2001) Hybrid genotypes in the pathogenic yeast *Cryptococcus* *neoformans*. *Microbiology* 147(Pt 4):891-907.

S8. Campbell LT, Currie BJ, Krockenberger M, Malik R, Meyer W, *et al.*, Clonality and recombination in genetically differentiated subgroups of *Cryptococcus* *gattii*. Eukaryot Cell 4: 1403-1409.

S9. Xu M, Huaracha E, Korban SS (2001) Development of sequence-characterized amplified regions (SCARs) from amplified fragment length polymorphism (AFLP) markers tightly linked to the Vf gene in apple. Genome 44: 63-70.

S10. Bovers M, Hagen F, Kuramae EE, Boekhout T, Six monophyletic lineages identified within *Cryptococcus* *neoformans* and *Cryptococcus* *gattii* by multi-locus sequence typing. Fungal Genet Biol 45: 400-421.

S11. D'Souza CA, Kronstad JW, Taylor G, Warren R, Yuen M, *et al*. (2011) Genome variation in *Cryptococcus* *gattii*, an emerging pathogen of immunocompetent hosts. mBio 2: e00342-10.

S12. Loftus BJ, Fung E, Roncaglia P, Rowley D, Amedeo P, *et al*. (2005) The genome of the basidiomycetous yeast and human pathogen *Cryptococcus* *neoformans*. Science 307: 1321-1324.

S13. Giegerich R, Meyer F, Schleiermacher C (1996) GeneFisher - software support for the detection of postulated genes. Proc Int Conf Intell Syst Mol Biol 8: 68-77.

S14. Byrnes III EJ, Li W, Lewit Y, Ma H, Voelz K, *et al*. (2010) Emergence and pathogenicity of highly virulent *Cryptococcus* *gattii* genotypes in the northwest United States. PLoS Pathog 6: e1000850.

S15. Illnait-Zaragozi MT, Martínez-Machín GF, Fernández-Andreu CM, Boekhout T, Meis JF, *et al*. (2010) Microsatellite typing of clinical and environmental *Cryptococcus* *neoformans* var. *grubii* isolates from Cuba shows multiple genetic lineages. PLoS One 5: e9124.

S16. Tamura K, Peterson D, Peterson N, Stecher G, Nei M, *et al*. (2011) MEGA5: Molecular Evolutionary Genetics Analysis using Maximum Likelihood, Evolutionary Distance, and Maximum Parsimony methods. Mol Biol Evol 28: 2731-2739.

S17. Simpson EH (1949) Measurement of diversity. Nature 163:688.

S18. Stoddart JA, Taylor JF (1988) Genotype diversity: estimation and prediction in samples. Genetics 118: 705-711.

S19. Meirmans PG, van Tienderen PH (2004) GenoType and GenoDive: two programs for the analysis of genetic diversity of asexual organisms. Mol Ecol Notes 4: 792-794.

S20. El Mousadik A, Petit RJ (1996) High level of genetic differentiation for allelic richness among populations of the argan tree [*Argania spinosa* (L.) Skeels] endemic to Morocco. Theor Appl Genet 92: 832-839.

S21. Goudet J (1995) FSTAT (version 1.2): a computer program to calculate F-statistics. J Hered 86: 485-486.

S22*.* Hudson RR (1987) Estimating the recombination parameter of a finite population model without selection. Genet Res 89: 427-432.

S23. Hudson RR, Kaplan NL (1985) Statistical properties of the number of recombination events in the history of a sample of DNA sequences. Genetics 111: 147-164.

S24. Librado P, Rozas J (2009) DnaSP v5: A software for comprehensive analysis of DNA polymorphism data. Bioinformatics 25: 1451-1452.

S25. Bruen TC, Philippe H, Bryant D (2006) A simple and robust statistical test for detecting the presence of recombination. Genetics 172: 2665-2681.

S26. van Iersel L, Kelk S, Rupp R, Huson DH (2010) Phylogenetic networks do not need to be complex: using fewer reticulations to represent conflicting clusters. Bioinformatics 26: i124-i131.

S27. Huson DH, Bryant D (2006) Application of phylogenetic networks in evolutionary studies. Mol Biol Evol 23: 254-267.

S28. Huson DH, Richter DC, Rausch C, Dezulian T, Franz M, et al. (2007) Dendroscope: An interactive viewer for large phylogenetic trees. BMC Bioinformatics 8: 460.

S29. Lyngsø RB, Song YS, Hein J (2005) Minimum recombination histories by branch and bound. Lecture Notes Bioinf 3692: 239-250.

S30. Tajima F (1989) DNA polymorphism in a subdivided population: the expected number of segregating sites in the two-subpopulation model. Genetics 123: 229-240.

S31. Fu YX, Li WH (1993) Statistical tests of neutrality of mutations. Genetics 133: 693-709.

S32. Price EW, Carbone I (2005) SNAP: workbench management tool for evolutionary population genetic analysis. Bioinformatics 21: 402-404.

S33*.* Beerli P, Felsenstein J (2001) Maximum Likelihood estimation of a migration matrix and effective population sizes in n subpopulations by using a coalescent approach. Proc Natl Acad Sci USA 98: 4563-4568.

S34. Bahlo M, Griffiths RC (2000) Inference from gene trees in a subdivided population. Theor Popul Biol 57: 79-95.

S35. Griffiths RC, Tavaré S (1994) Sampling theory for neutral alleles in a varying environment. Philos Trans R Soc Lond B Biol Sci 344: 403-410.

S36. Sharpton TJ, Neafsey DE, Galagan JE, Taylor JW (2008) Mechanisms of intron gain and loss in *Cryptococcus*. Genome Biol 9: R24.

S37. Botes A, Boekhout T, Hagen F, Vismer H, Swart J, *et al*. (2009) Growth and mating of *Cryptococcus* *neoformans* var. *grubii* on woody debris. Microb Ecol 57: 757-765.

S38. Ishaq CM, Bulmer GS, Felton FG (1968) An evaluation of various environmental factors affecting the propagation of *Cryptococcus neoformans*. Mycopathol Mycol Appl 35: 81-90.

S39. Kidd SE, Chow Y, Mak S, Bach PJ, Chen H, *et al*. (2007) Characterization of environmental sources of the human and animal pathogen *Cryptococcus* *gattii* in British Columbia, Canada, and the Pacific Northwest of the United States. Appl Environ Microbiol 73: 1433-1443.

S40. Chevreux B, Wetter T, Suhai S (1999) Genome sequence assembly using trace signals and additional sequence information. Comp Sci Biol Proc Germ Conf Bioinf 99: 45-56.

S41. Stanke M, Schöffmann O, Morgenstern B, Waack S (2006) Gene prediction in eukaryotes with a generalized hidden Markov model that uses hints from external sources. BMC Bioinf 7: 62.

S42. Langmead B, Salzberg S. (2012) Fast gapped-read alignment with Bowtie 2. Nature Methods 9: 357-359.

S43*.* McKenna A, Hanna M, Banks E, Sivachenko A, Cibulskis K, *et al.* (2010) The Genome Analysis Toolkit: a MapReduce framework for analyzing next-generation DNA sequencing data. Genome Res 20: 1297-1303.

S44*.* Altschul SF, Gish W, Miller W, Myers EW, Lipman DJ (1990) Basic local alignment search tool. J Mol Biol 215: 403-410.

S45. Edgar RC (2004) MUSCLE: multiple sequence alignment with high accuracy and high throughput. Nucleic Acids Res 32: 1792-1797.

S46. Capella-Gutiérrez S, Silla-Martínez JM, Gabaldón T (2009) TrimAl: a tool for automated alignment trimming in large-scale phylogenetic analyses. Bioinformatics 25: 1972-1973.

S47. Jukes TH, Cantor CR (1969) in *Evolution of Protein Molecules* (Academic Press, New York), pp. 21-132.

**Figure S1: Microsatellite analysis of *C. gattii* AFLP6/VGII strains.** Minimum spanning tree analysis based on ten microsatellite loci showing the genetic diversity of the complete set of *C. gattii* AFLP6/VGII strains and especially that of the South American population (red). Case cluster and outbreak related clusters are indicated. Colours represents the populations of Africa (yellow), Australasia (blue), Europe (purple), North America (green) and South America (red). Circle sizes are relative to the number of strains that are represented by that microsatellite type. Connecting lines between circles indicate the number of different microsatellite between both groups. Thick black lines represent one different locus, thin black two loci, dashed black line three loci, dashed dark grey line four loci and five or more different loci are indicated with dashed light gray lines plus number of different loci.

**Figure S2: Neighbour Joining analysis of arbitrarily scored AFLP markers.** The matrix of absent (0) and present (1) differential AFLP markers (Table S3) has been used to generate a Neighbour Joining tree including an outgroup of *C. neoformans* and *C. gattii* reference strains. Haplotype numbers are provided as shown in the coalescence gene genealogy and populations are indicated behind the strain numbers as AFR (Africa), AUS (Australasia), EUR (Europe), NA (North America) and SA (South America).

**Figure S3: Phylogenetic analysis of the extended ‘Fraser & Byrnes’ MLST dataset.** Unrooted Maximum Likelihood phylogenetic analysis of the sequence types found within the concatenated dataset of seven nuclear MLST loci as provided by Byrnes et al. [S14] and Fraser et al. [S4] and extended with additional strains in the current study. The most ancestral lineage (here ST07) from the SCAR-MLST based coalescence gene genealogy analysis is indicated in this figure with a tree to highlight its origin from pristine Amazon rainforest. When rooted with *C. gattii* genotype AFLP4/VGI, AFLP5/VGIII and AFLP7/VGIV, strains within ST18 are basal to the other lineages. The outbreaks in North America are indicated with icons of human, cat and dolphin, and the parrot in the South American population highlight the case cluster of *C. gattii* infections among psittacine birds in Southern Brazil. Populations are indicated behind strain and haplotype numbers as AFR (Africa), AUS (Australasia), EUR (Europe), NA (North America) and SA (South America).

**Figure S4: Ancestral recombination graph analysis based on SCAR-MLST.** The ancestral recombination graph shows historical recombination events that gave rise to the current population structure represented by the clone corrected 49 sequence types (STs) observed within the concatenated nuclear SCAR-MLST dataset (Table S3). The ST01/ST02 lineage represents strains with the major Vancouver Island outbreak genotype AFLP6A/VGIIa (indicated with a human, cat and dolphin symbol to represent the clinical and veterinary strains), similarly indicated is ST04 representing the minor genotype AFLP6B/VGIIb. The recently emerged *C. gattii* AFLP6C/VGIIc Pacific Northwest outbreak (ST39) has been indicated with a human and cat symbol. The tree icon indicates the most ancestral lineage that was isolated from a tree in the pristine Amazon rainforest (ST26). The 167 blue circles indicate historical recombination breakpoint positions within the concatenated nuclear SCAR-MLST dataset.

**Figure S5: CASS network analysis based on SCAR-MLST.** Recombination network analysis based on the CASS algorithm shows that several recombination events occurred in the current global population and that the majority of parental donors came from the South American population. Populations are indicated behind strain and haplotype numbers as AFR (Africa), AUS (Australasia), EUR (Europe), NA (North America) and SA (South America). The outbreaks in North America are indicated with icons of human, cat and dolphin, and the parrot-icon in the South American population highlight the case cluster of *C. gattii* infections among psittacine birds in Southern Brazil.

**Figure S6: Growth curves of *C. gattii* AFLP6/VGII strains on *myo*-inositol.** Growth curves of the three replicated growth rate experiments for A1M-R265 (major genotype AFLP6A/VGIIa Vancouver Island outbreak lineage) and CBS1930 (Aruba). The formula for each of the trendlines is provided as this is part of the formula to calculate the generation time. For the coalescence gene genealogy generation time scale calculation, the average value is used from all six growth rate experiments.

**Table S1: Map of informative sites used for the coalescence gene genealogy analysis.** The clone corrected SCAR-MLST data has been collapsed into haplotypes after removal of homoplasious sites. Informative sites within the complete dataset, and among the 32 haplotypes identified, are provided per nuclear SCAR-MLST locus that has been provided as the Fragment-number. IGS1 refers to the Intergenic Spacer 1 region (see Table S5). Colours used for each of the loci correspond with those used to mark the mutation events along the coalescence gene genealogy in Fig. 1.

**Table S2: Population sizes and historical migration rate estimates among *Cryptococcus gattii* populations.** The population sizes and migration rates were estimated using Migrate v2.3 ([http://popgen.scs.fsu.edu](http://popgen.scs.fsu.edu/)) based on the five nuclear SCAR-MLST loci. The centre column shows the mean Nmµ value, while the left flanking values represents the lower (left value) and upper (right value) 95% confidence interval values.

**Table S3: List of strains and background information.**

**Table S4: Overview of molecular variation, including number of haplotypes, nucleotide diversity, estimates of theta based on the number of segregating sites and recombination parameters**. The genetic diversity is provided per population and for all strains, as well as for each locus independently and the mean value for the given values. Given values are for the number of sites within an alignment with or without alignment gaps, the nucleotide diversity per site (π) corresponding to the average number of nucleotide differences per site between two sequences, the number of segregating or polymorphic sites (S), the Watterson’s estimated θ per locus and per nucleotide, the ‘Hud4Nc per site’ value representing the recombination rate per generation between the most distant nucleotides. The last two values listed the number of recombination events based on the ‘four gametic test’ and the minimum number of recombination events in the history of the sample (Note that provides an underestimation of the number of recombination events).

**Table S5:** Primers used for SCAR-MLST and microsatellite typing.
